# Sirt1 regulates glial progenitor proliferation and regeneration in white matter after neonatal brain injury

**DOI:** 10.1038/ncomms13866

**Published:** 2016-12-19

**Authors:** Beata Jablonska, Marcin Gierdalski, Li-Jin Chew, Teresa Hawley, Mackenzie Catron, Arturo Lichauco, Juan Cabrera-Luque, Tracy Yuen, David Rowitch, Vittorio Gallo

**Affiliations:** 1 Center for Neuroscience Research, Children's National Medical Center, 111 Michigan Avenue, Washington, District Of Columbia 20010, USA; 2 Division of Cardiac Surgery, Children's National Medical Center, 111 Michigan Avenue, Washington, District Of Columbia 20010, USA; 3 Flow Cytometry Core Facility, George Washington University, Washington, District Of Columbia 20052, USA; 4 Center for Genetic Medicine, Children's National Medical Center, 111 Michigan Avenue, Washington, District Of Columbia 20010, USA; 5 Department of Pediatrics, Eli and Edythe Broad Institute for Stem Cell Research and Regeneration Medicine, University of California, San Francisco, San Francisco, California 94143, USA; 6 Howard Hughes Medical Institute, University of California, San Francisco, San Francisco, California 94143, USA

## Abstract

Regenerative processes in brain pathologies require the production of distinct neural cell populations from endogenous progenitor cells. We have previously demonstrated that oligodendrocyte progenitor cell (OPC) proliferation is crucial for oligodendrocyte (OL) regeneration in a mouse model of neonatal hypoxia (HX) that reproduces diffuse white matter injury (DWMI) of premature infants. Here we identify the histone deacetylase Sirt1 as a Cdk2 regulator in OPC proliferation and response to HX. HX enhances Sirt1 and Sirt1/Cdk2 complex formation through HIF1α activation. Sirt1 deacetylates retinoblastoma (Rb) in the Rb/E2F1 complex, leading to dissociation of E2F1 and enhanced OPC proliferation. Sirt1 knockdown in culture and its targeted ablation *in vivo* suppresses basal and HX-induced OPC proliferation. Inhibition of Sirt1 also promotes OPC differentiation after HX. Our results indicate that Sirt1 is an essential regulator of OPC proliferation and OL regeneration after neonatal brain injury. Therefore, enhancing Sirt1 activity may promote OL recovery after DWMI.

## Abstract (web summary)

Oligodendrocyte progenitor cell (OPC) proliferation is crucial for regeneration after hypoxic lesions in mice, a model of diffuse white matter injury of premature infants. Here, the authors show that the histone deacetylase Sirt1 is a Cdk2-dependent mediator of OPC proliferation and OPC response to hypoxia.

Cell regeneration observed in brain injury and in a variety of neurological disorders—including hypoxia (HX)/ischaemia, demyelination, stroke, spinal cord injury, and Huntington's or Alzheimer's disease—depends on endogenous progenitor cells that respond to various insults by expansion of their pools^1^,^2^. HX is a major cause of diffuse white matter injury (DWMI), which is associated with permanent developmental disabilities in prematurely born infants^3^,^4^,^5^,^6^. DWMI is characterized by altered development and long-term abnormalities of the white matter, caused by oligodendrocyte (OL) loss and delayed functional myelination^7^,^8^,^9^. Proliferating OL progenitor cells (OPCs) are the main source of newly generated OLs and are capable of repopulating injured white matter regions, leading to myelin regeneration and functional recovery^10^,^11^. Current therapies for DWMI are still not curative, therefore it is crucial to target endogenous OPCs and enhance their expansion after injury to maximize white matter repair.

Using a mouse model of neonatal HX that reproduces morphological and structural brain abnormalities found in DWMI of prematurely born infants^12^,^13^,^14^,^15^,^16^, we previously demonstrated that HX triggers a regenerative response in OPCs that involves enhanced proliferation through activation of the Cdk2 pathway, and delayed differentiation caused by reduced levels of p27^Kip1^ (ref. 14). However, the molecular pathways that play a crucial role in coupling HX to enhanced OPC proliferation are still unknown. Defining these molecular mechanisms in DWMI is of crucial importance to reactivate intrinsic developmental pathways functionally involved in OL regeneration and ultimately in white matter recovery. Furthermore, these mechanisms might be relevant to a variety of pathologies of the developing central nervous system, as the regenerative response of neural progenitors to injury in the immature brain is largely unexplored.

The nicotinamide adenine dinucleotide (NAD)-dependent class III histone deacetylase (HDAC) Sirt1 is involved in normal cell development and fate determination, as well as in ageing, inflammatory responses and energy metabolism^17^,^18^,^19^,^20^. Among many different roles in metabolism and calorie restriction, Sirt1—as a sensor of redox status in cells—is also involved in the response to environmental stress modulated by HX through deacetylation of hypoxia-inducible factor 1α (HIF1α)^21^,^22^,^23^. Sirt1 is also involved in modulating the activity of cell cycle regulatory proteins, as this is determined by their phosphorylation and acetylation state. Cyclin-dependent kinases (Cdks)—which are positively regulated by their regulatory subunits (cyclins, Cyc)—phosphorylate members of the pocket protein family (Rb, p107 and p130)^24^. In turn, the acetylation state of both Cdks and pocket proteins is regulated by HDACs, including Sirt1 (ref. 25).

Sirt1, when engaged in mitotic cell activity^26^, is transcriptionally regulated by p53, E2F1, FoxO3a and the HIC1–CtBP complex^27^, and undergoes a variety of post-translational modifications^28^. Sirt1 deacetylase activity is also regulated by formation of the Sirt1/Cdk1/Cyc B complex and subsequent Sirt1 phosphorylation by Cdk1 (ref. 26). Conversely, Sirt1 deacetylates a member of the Cdk2 pathway, the retinoblastoma (Rb) protein^29^, which plays a crucial functional role in G1–S transition of the cell cycle.

A recent report demonstrated that Sirt1 maintains mouse embryonic stem cells in an undifferentiated/self-renewing state, particularly under oxidative conditions^18^, suggesting that Sirt1 might play an important role in self-renewal and proliferation of progenitor/stem cells. The role of Sirt1 in neural progenitor proliferation in early postnatal brain development—in particular in response to injury—has not been defined. Furthermore, it has not been determined whether Sirt1 modifies neural progenitor cell cycle activity through deacetylation of individual members of the Cdk2/Rb/E2F1 complex.

In the present study, we investigated the roles of Sirt1 in immature neural cell proliferation, and as a deacetylase in HIF1α-regulated pathways in the context of early postnatal OPC response to HX. We identify Sirt1 as a novel, major regulator of basal OPC proliferation and regeneration in response to HX in neonatal white matter. We demonstrate Sirt1 phosphorylation by Cdk2, and also elucidate the mechanism by which Sirt1 targets individual members of the Cdk2 signalling pathway, regulating their deacetylation, complex formation and E2F1 release, molecular events, which drive Cdk2-mediated OPC proliferation^14^,^30^.

## Results

### Neonatal HX regulates Sirt1 expression in parenchymal OPCs

We have previously demonstrated that neonatal HX enhances proliferation of two distinct pools of glial progenitors in a time-dependent manner^14^. HX (from P3 until P11) induces proliferation of white matter NG2-expressing (NG2^+^) OPCs for a week after the insult, conversely proliferation of SVZ NG2^+^ cells continues for at least a month^14^. Since white matter development is drastically disrupted after neonatal brain injury^16^, we focused on the regenerative response of parenchymal white matter OPCs after HX. We have previously shown that Cdk2 plays an important role in regulating basal and HX-induced OPC proliferation^14^, but the molecular mechanisms involved are still undefined. It has been demonstrated that many cell cycle proteins that control cell proliferation are sirtuin substrates^31^, and sirtuins—including Sirt1—regulate proliferation of different cell types^31^. Therefore, we focused on the role of sirtuins, particularly Sirt1, in regulating OPC proliferation after HX.

**Figure 1:**
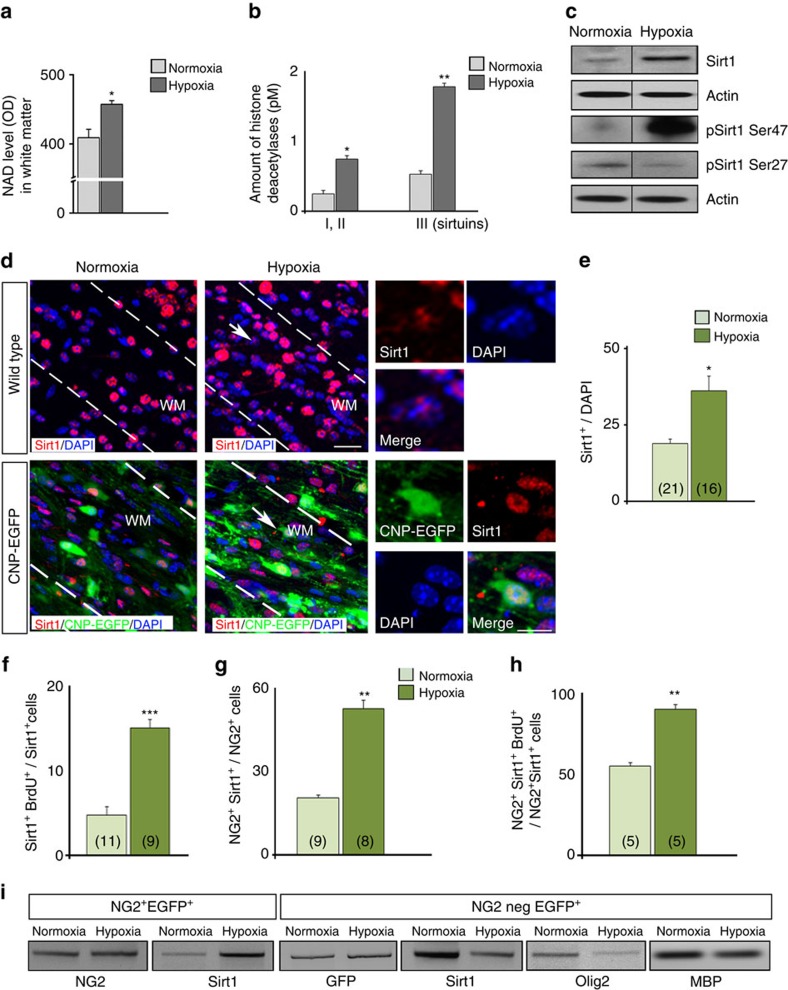
Neonatal HX increases Sirt1 expression in OPCs of the white matter. (**a**) NAD level in white matter after NX and HX expressed in optical density (OD) units. Mean±s.e.m., four independent experiments, two brains per condition measured in triplicate. (**b**) Levels of histone deacetylases class I+II and III in white matter in NX and HX expressed in pmol l^−1^ (pM). Mean±s.e.m., two independent experiments, three brains per condition in triplicate. (**c**) Representative western blot of Sirt1, and pSirt1 Ser47 and pSirt1 Ser27 in NX and HX white matter (P18).(**d**) Confocal images of Sirt1^+^ cells in NX and HX white matter in WT and CNP-EGFP mice. Dotted lines delineate white matter. WM, white matter. Scale bar, 100 μm. Arrows indicate magnified cells. HX increases Sirt1^+^ cells (**e**), proliferating Sirt1^+^ (**f**), NG2^+^Sirt1^+^ (**g**) and NG2^+^Sirt1^+^BrdU^+^ (**h**) cells in white matter. Mean±s.e.m. Number of samples indicated in parentheses, *n*=4 brains per condition. (**i**) Semi-quantitative RT–PCR analysis of *Sirt1*, *Olig2* and *MBP mRNAs* from purified NG2^+^EGFP^+^ progenitors and NG2negEGFP^+^ OLs.

It is known that NAD regulates Sirt1 activity, and serves as a major coenzyme in redox reactions^32^. Significant alterations of the cellular redox status have been demonstrated during both self-renewal and cell differentiation^33^. To study whether HX modifies levels of the intracellular nucleotide NAD, we performed nicotinamide assay and demonstrated higher levels of NAD in white matter cells after HX (Fig. 1a).

To determine the specific contribution of different classes of HDACs to the cellular changes induced by HX, we performed a fluorometric HDAC activity assay in normoxic (NX) and HX white matter tissue. The activity of all HDACs was significantly higher after HX (Fig. 1b), however the relative increase in sirtuins (class III deacetylases) activity was much higher than the increase observed in HDACs I and II (Fig. 1b). In summary, our results obtained in cultured cells and in tissue strongly suggest that Sirt1 could be a possible mediator of the effects of HX on OPC redox state during cell cycle progression.

To determine whether enhanced NAD levels play a role in class III deacetylase activation after HX, we performed *in vitro* experiment, in which NX and HX cells were cultured in control medium, or in the presence of NADH or NAD. We hypothesized that NAD promotes deacetylation of Cdk2 through sirtuins, particularly Sirt1; therefore, we immunoprecipitated Cdk2 to determine its acetylation status. Western blot analysis revealed lower levels of acetylated Cdk2 after HX in control medium, indicating increased deacetylation. In the presence of NADH, deacetylated Cdk2 levels were similar in NX and HX lysates. Conversely, exposure of the cultures to NAD markedly enhanced Cdk2 deacetylation (Supplementary Fig. 1a,b). To determine whether NAD-stimulated deacetylation of Cdk2 was mediated by Sirt1, we knocked down Sirt1 expression in cultured NX and HX cells after exposure to high concentrations of NAD. Western blot analysis revealed higher levels of acetylated Cdk2 after Sirt1 knockdown, indicating that Sirt1 is the primary deacetylase of Cdk2 under conditions of HX and increased NAD levels (Supplementary Fig. 1c). Cdk2 deacetylation is likely to induce its kinase activity, given that acetylation by the acetyltransferase P300/CBP-associated factor (PCAF) has been shown to inactivate Cdk2 kinase activity^34^).

We next determined whether HX induced changes in Sirt1 expression in white matter *in vivo*. Western blot analysis on whole lysates from NX and HX white matter revealed higher levels of total Sirt1 expression after HX (Fig. 1c). Previous analysis identified 13 amino-acid residues—mainly at serine (Ser) and threonine (Thr)—as potential Sirt1 phosphorylation sites^28^. We focused on two of these sites (Ser27 and Ser47), as they match minimal consensus sequences of Cdk phosphorylation substrates, which are believed to stabilize the level of Sirt1 protein^28^. Western blot analysis revealed that HX also enhanced Sirt1 phosphorylation at Ser47 and downregulated Ser27 phosphorylation (Fig. 1c). Higher expression of pSirt1 Ser47 may reflect direct phosphorylation of this site by Cdks, possibly by Cdk2.

Consistent with enhanced expression of total Sirt1 protein levels in white matter, HX also increased the percentage of Sirt1^+^ cells in white matter (Fig. 1d,e). We have previously demonstrated that in the 2′,3′-cyclic-nucleotide 3′-phosphodiesterase (CNP)-EGFP mouse, in which all developmental stages of the OL lineage can be visualized based on EGFP expression, the majority of proliferating EGFP^+^ cells under normal or pathological conditions are OPCs^14^,^35^. Therefore, we wanted to determine whether Sirt1 expression was upregulated in proliferative OPCs. Cellular analysis in white matter of CNP-EGFP mice revealed that HX increased Sirt1 expression in both NG2^+^ and NG2^+^BrdU^+^ OPCs (Fig. 1f–h). To determine whether HX-induced changes in Sirt1 occurred at the transcriptional level, we analysed relative levels of *Sirt1* mRNA transcripts in NG2^+^EGFP^+^ OPCs fluorescence-activated cell sorting (FACS)-purified from NX and HX white matter. Higher levels of *Sirt1* mRNAs were detected after HX in this cell population (Fig. 1i); conversely, *Sirt1* transcripts were downregulated in NG2-negative EGFP^+^ OLs, along with lower expression levels of *Olig2* and *MBP* mRNAs (Fig. 1i).

Further cellular analysis of white matter in wild-type (WT) and CNP-EGFP mice showed that although HX elevated the total percentage and proliferation of NG2^+^ and Mash1^+^ progenitor cells (Supplementary Fig. 2a–f), Sirt1 expression was enhanced only in NG2^+^, but not in Mash1^+^ cells (Supplementary Fig. 2g). Similar effects of HX on Sirt1 expression in glial progenitors were also observed in the SVZ (Supplementary Fig. 3). Finally, no change in the numbers of GFAP^+^ astrocytes, CC1^+^ mature OLs and Iba1^+^ microglia that expressed Sirt1 was observed in white matter (Supplementary Fig. 4). Altogether, these results indicate that HX upregulates Sirt1 expression and phosphorylation in the developing white matter, particularly in proliferating OPCs expressing NG2. In addition, HX does not alter Sirt1 expression in other glial cell types.

To determine whether Sirt1 plays a specific role in OPC proliferation in neonatal white matter, we used a model of focal (lysolecithin-induced) demyelination of adult white matter^35^. We analysed white matter lesions at 7 days after demyelination, that is, at a time point when OPC proliferation is maximal^35^. Differently from neonatal HX, focal demyelination led to a reduction of Sirt1^+^ and Sirt1^+^BrdU^+^ cells in the adult white matter (Supplementary Fig. 5a–c). Demyelination also reduced the percentage of NG2^+^ progenitors expressing Sirt1, as well as the percentage of proliferating Sirt1^+^NG2^+^ cells (Supplementary Fig. 5d,e). Consistent with previously published data^35^, enhanced astrocyte and microglial cell numbers were found in the lesioned area (Supplementary Fig. 5f,g), however no changes in Sirt1 expression were detected in these cells (Supplementary Fig. 5f,i,j). Finally, we also observed lower percentages of CC1^+^ mature OLs and CC1^+^Sirt1^+^ cells after focal demyelination (Supplementary Fig. 5f–h). Altogether, these results demonstrate that focal demyelination of adult white matter causes opposite effects on Sirt1 expression compared with HX, indicating that enhanced Sirt1 expression observed after neonatal HX is specific for this type of injury.

**Figure 2:**
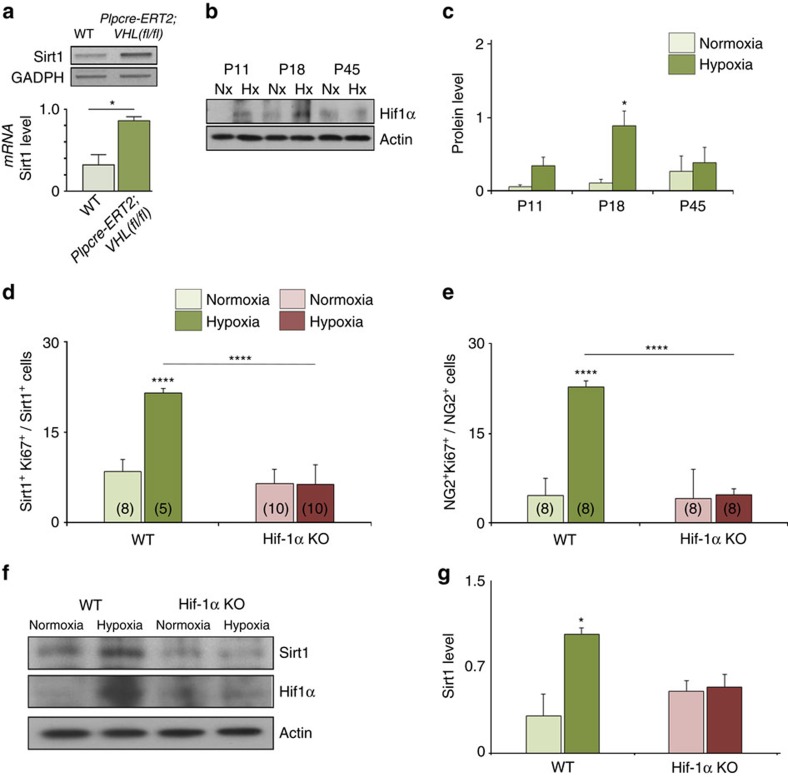
HX-induced Sirt1 expression in white matter OPCs requires HIF1α. (**a**) HIF1α stabilization of Sirt1 transcript expression in OPCs as revealed by representative RT–PCR represents higher level of Sirt1 *mRNA* in *VHL* cKO mice. *GDPDH mRNA* serves as a control. Mean±s.e.m., *n*=3 brains for each group. (**b**,**c**) Representative western blot demonstrates a transient increase of HIF1α expression in HX white matter at P18 with no significant effect at P11 (*P*=0.7955) and P45 (*P*=0.7333). Histograms show mean±s.e.m. (**d**,**e**) Graphs represent the percentages of Sirt1^+^Ki67^+^ and NG2^+^Ki67^+^ cells after HX white matter in WT and HIF1α KO mice. Number in parentheses within bar indicates number of samples (*n*=4 brains per group and per genotype; ****P<0.0001, one-way analysis of variance, Bonferroni *post hoc* test, mean±s.e.m.). (**f**,**g**) Western blot demonstrates no increase (*P*=0.8231) in Sirt1 and HIF1α expression in white matter of Hif1α KO mice. Actin was used as loading control (mean±s.e.m.; *n*=3 brains for each experiment, group and genotype).

It has been previously demonstrated that HIF1α regulates Sirt1 transcriptional expression *in vitro* and *in vivo*^21^. HIF1α is hydroxylated by specific prolyl-hydroxylases and targeted for rapid proteasomal degradation by the von Hippel–Lindau (VHL) tumour suppressor^36^. Therefore, to examine the effect of HIF1α stabilization on Sirt1 expression, we selectively ablated *VHL* expression in OPCs and purified these cells from *Plpcre-ERT2;VHL*(*fl/fl*) (*VHL* cKO) neonatal brains by immunopanning^37^. Comparing OPCs isolated from *VHL* cKO mice with and without treatment with tamoxifen, we found that HIF1α stabilization enhanced Sirt1 expression, as shown by reverse transcription–PCR (RT–PCR; Fig. 2a). Next, we determined whether HX-induced regulation of Sirt1 protein in OPCs depended on HIF1α. Immunoblotting of Hif1α revealed a time-dependent increase in protein level with a peak of expression at 1 week after HX (P18), followed by a decrease at later stages (P45; Fig. 2b,c). In white matter of Hif1α knockout mice (*HIF1α* KO) exposed to HX, we found a significantly lower number of proliferating Sirt1^+^ cells at P18 (Fig. 2d), as well as proliferating NG2^+^ progenitors (Fig. 2e). Finally, in the absence of Hif1α, HX had no effect on Sirt1 expression in white matter as determined by western blot (Fig. 2f,g). In these experiments, HIF1α ablation had no effect on basal Sirt1 expression under NX. Altogether, these findings demonstrate that (i) HX-induced Sirt1 expression in OPCs depends on the presence of Hif1α and (ii) HX regulates Hif1α expression in a time-dependent manner.

### Sirt1 regulates OPC proliferation in cultured cells

**Figure 3:**
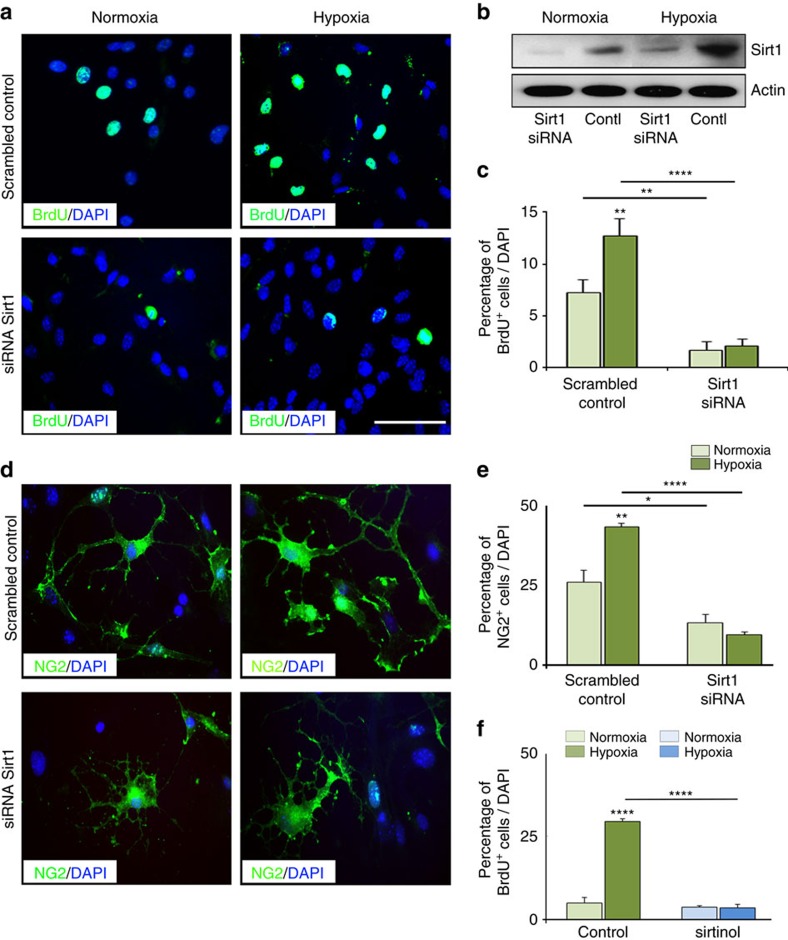
Sirt1 knockdown reduces proliferation of cultured OPCs after HX. NX and HX white matter (P18) cells were transfected with Sirt1 siRNA and scrambled control, and labelled with BrdU (**a**) and NG2 (**d**). Scale bar, 100 μm. (**b**) Representative western blot analysis demonstrates Sirt1 knockdown in transfected NX and HX cells, compared with cells transfected with scrambled control (*n*=3 brains per group, per treatment). Sirt1 siRNA treatment shows a reduction in the percentage of BrdU^+^ cells (**c**) and NG2^+^ cells (**e**) in both NX (basal proliferation) and HX compared with controls. (**f**) Inhibition of Sirt1 activity by sirtinol diminished overall cell proliferation after HX (five NX and six HX brains for each treatment; four cultures for each conditions; *P<0.05; **P<0.01; ****P<0.0001, one-way analysis of variance, Bonferroni *post hoc* test, mean±s.e.m.).

The changes in Sirt1 expression observed in OPCs after HX suggest a functional involvement of this HDAC in HX-induced OPC proliferation in the developing white matter. To investigate a possible functional role of Sirt1 in cell proliferation after neonatal brain injury, we first performed a knockdown of Sirt1 expression in cultured white matter cells obtained from NX and HX mice. Western blot analysis showed that siRNA-mediated knockdown resulted in more than 90% decrease in Sirt1 expression in both NX and HX cells (Fig. 3a,b). Sirt1 knockdown resulted in a decrease in basal OPC proliferation from 7.32±1.3 to 1.56±0.6% (mean±s.e.m.; Fig. 3c). HX caused a twofold increase in the percentage of BrdU^+^ cells in cultures treated with a scrambled control siRNA (Fig. 3c), but no effects in cultures transfected with Sirt1 siRNA (Fig. 3c). Furthermore, the increase in percentage of NG2^+^ cells observed after HX was prevented by Sirt1 siRNA treatment (Fig. 3d,e). We then used sirtinol, a strong inhibitor of Sirt1 activity^38^,^39^, to further demonstrate a role for Sirt1 in OPC proliferation after HX. Treatment of cultured OPCs with sirtinol abolished the effects of HX on proliferation (Fig. 3f). Altogether, both the siRNA and sirtinol experiments consistently point to Sirt1 as an important regulator of basal and HX-induced OPC proliferation.

To determine whether Sirt1 plays a specific role in white matter OPC proliferation after HX, we also investigated the role of Sirt1 in cultured SVZ OPCs. Differently from white matter OPCs, treatment with Sirt1 siRNA did not modify basal or HX-induced changes in SVZ OPC proliferation and number (Supplementary Fig. 6). These results indicate that Sirt1 plays a white matter-specific role in regulating basal and HX-induced OPC proliferation.

**Figure 4:**
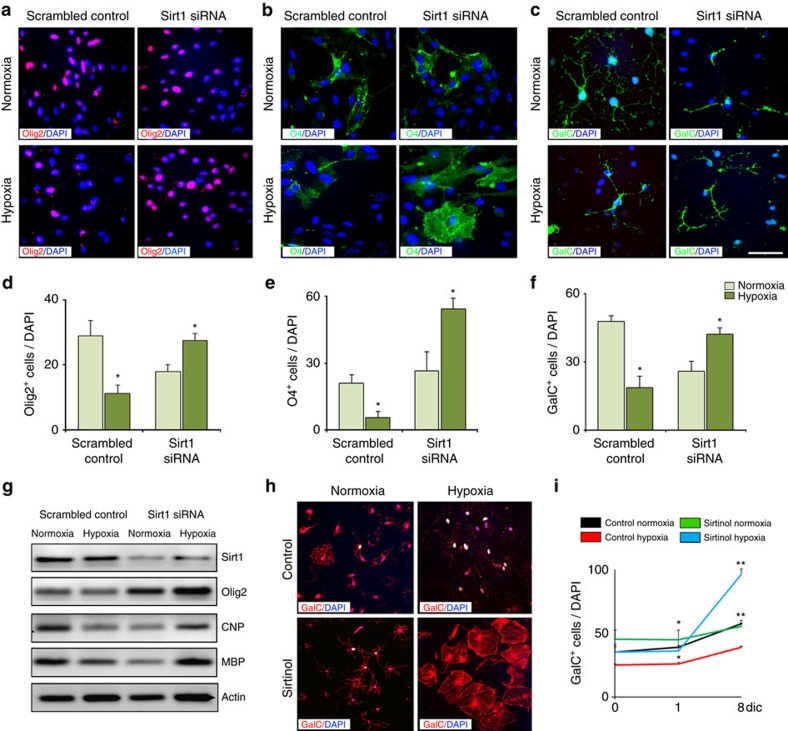
Sirt1 knockdown enhances OL differentiation in cultured white matter cells after neonatal HX. NX and HX cells transfected with Sirt1 siRNA and scrambled control, and labelled with anti-Olig2 (**a**), -O4 (**b**) and -GalC (**c**) antibodies. Scale bar, 100 μm. Decreased percentages of Olig2^+^ (**d**), O4^+^ (**e**) and GalC^+^ cells (**f**) in control cultures after HX, and significantly increased OLs after Sirt1 siRNA (five NX and six HX brains per group; *P<0.05, one-way analysis of variance (ANOVA), Tukey's *post hoc* test, mean±s.e.m.). (**g**) Representative RT–PCR from NX and HX cells treated as above. *Olig2*, *CNP* and *MBP* mRNA expressions are reduced in controls and elevated in Sirt1 siRNA cultures. Actin serves as control (*n*=3 brains for each condition, each treatment). (**h**) Sirtinol increases OPC differentiation only in HX cultures. (**i**) Graph represents increase of GalC^+^ cells after sirtinol treatment (five NX and six HX brains for each treatment; *P<0.05, **P<0.01, one-way ANOVA, Tukey's *post hoc* test, mean±s.e.m.).

Finally, we also assessed the consequences of Sirt1 inhibition on OPC differentiation in culture by immunolabelling with OL markers, including Olig2, O4 and GalC. In HX cells treated with scrambled control, a reduction in Olig2^+^ OL lineage cells was observed, as well as a decrease in O4^+^ pre-OLs and in GalC^+^ mature OLs (Fig. 4a–f). siRNA-mediated Sirt1 inactivation increased the percentage of cells expressing Olig2, O4 and GalC (Fig. 4a–f). Consistent with these findings, higher levels of *Olig2*, *CNP* and *MBP mRNA* transcripts were also detected in HX cultures after siRNA-mediated Sirt1 inactivation (Fig. 4g). In addition, sirtinol treatment also promoted cell differentiation in HX cultures, but had no effects on NX cells (Fig. 4h,i). These data indicate that Sirt1 activity in OPCs regulates the balance between OPC proliferation and differentiation after HX.

### Sirt1 regulates OPC proliferation *in vivo*

To study the functional role of Sirt1 in OPC development after HX *in vivo*, we generated an inducible Sirt1 knockout mouse strain (*Sirt1*^*F/F*^*;PDGFR*^*CreER*^*;Rosa*^*YFP*^, *Sirt1*-cKO) in which *Sirt1* is selectively inactivated in OPCs after tamoxifen injection. We first compared tamoxifen-injected *Sirt1-*cKO mice with those which were not injected. Tamoxifen induced *Cre* recombination and reduced Sirt1 expression in white matter cells (Supplementary Fig. 7a,b). We then compared tamoxifen-injected *Sirt1-*cKO mice with tamoxifen-injected WT littermates. Reduced Sirt1 expression was observed in white matter cells in *Sirt1-*cKO mice, as compared with WT (Supplementary Fig. 7c,d). Importantly, virtually no *PDGFR*^*CreER*^ cells displayed Sirt1 expression (Supplementary Fig. 7d).

**Figure 5:**
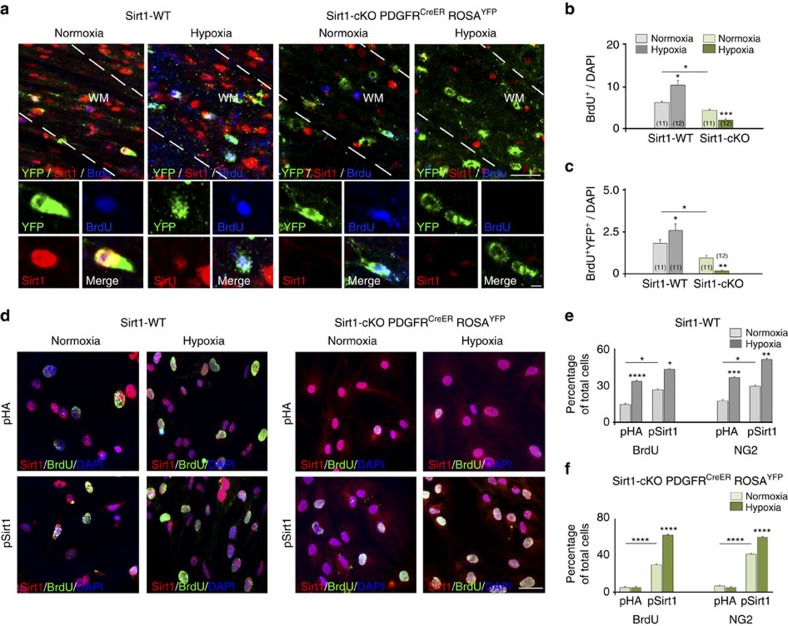
Targeted ablation of *Sirt1* in OPCs abolishes HX-induced proliferation in white matter. (**a**) Confocal images from Sirt1-WT and *Sirt1-*cKO mice after NX and HX. Cells were immunolabelled with anti-Sirt1, -GFP (to detect YFP) and -BrdU antibodies. Dotted lines delineate white matter (WM). Scale bar, 100 μm. (**b**,**c**) Graphs represent reduced proliferation in NX white matter of *Sirt1-*cKO mice compared with Sirt1-WT (**b**). HX increases percentages of BrdU^+^ and BrdU^+^YFP^+^ cells in Sirt1-WT mice, and decreases in *Sirt1-*cKO (**b**,**c**). Histograms show mean±s.e.m. Number of samples indicated in parentheses, 4 brains per condition and genotype. (**d**–**f**) Overexpression with pSirt1 plasmid in NX and HX white matter cells from Sirt1-WT and *Sirt1-*cKO (P18). Cells stained with anti-Sirt1, -BrdU antibodies and DAPI. Scale bar, 50 μm. (**e**,**f**) pSirt1 increased percentage of BrdU^+^ and NG2^+^ cells in NX cultures versus pHA controls. In *Sirt1-*cKO cultures, pSirt1 restored HX-induced BrdU^+^ and NG2^+^ cells. Histograms show mean±s.e.m. (three NX brains and four HX brains per genotype; four cultures per condition).

In conditional *Sirt1-*cKO mice, we observed a reduced percentage of proliferating cells in NX white matter compared with WT (5.92±0.75%—WT littermates; 3.62±0.23%—*Sirt1*-cKO, mean±s.e.m.; Fig. 5a–c). Gain-of-function experiments, in which cultured NX cells were transfected with either control or *Sirt1* plasmid showed an increase in proliferation from 15.33±2.56 to 30.50±1.6%, mean±s.e.m. (Fig. 5d,e). Moreover, decreased cell proliferation observed in OPC-specific *Sirt1* knockout (from 15.33±2.6 to 6.6±0.5%, mean±s.e.m.; Fig. 5e,f) was rescued by incubation with *Sirt1*-expressing plasmid (29.78±07%, mean±s.e.m.; Fig. 5f). These results support the idea that Sirt1 also regulates basal OPC proliferation.

To determine if Sirt1 regulates white matter OPC proliferation after HX *in vivo*, we first compared cell proliferation in WT and *Sirt1-*cKO mice. HX-induced OPC proliferation was completely prevented by Sirt1 inactivation in OPCs (Fig. 5a–c). Almost no proliferating OPCs were found in white matter of *Sirt1-*cKO mice after HX (Fig. 5c), indicating a major role for Sirt1 in regulating proliferation of PDGFRα-expressing OPCs after HX *in vivo*.

Next we determined whether Sirt1 overexpression in white matter OPCs rescued their proliferative potential after HX. Therefore, we overexpressed Sirt1 in white matter cells cultured from WT and *Sirt1-*cKO mice. In WT cultures, HX enhanced cell proliferation and the percentage of NG2^+^ progenitor cells after transfection with either a control (pHA) plasmid or after transfection with a pSirt1 plasmid (Fig. 5d,e). Conversely, in cells isolated from *Sirt1-*cKO mice and transfected with the control pHA plasmid, HX had no effects on the percentage of BrdU^+^ and NG2^+^ cells (Fig. 5d,f). Basal cell proliferation was restored in NX cells after Sirt1 rescue, and so was the effect of HX on cell proliferation and percentage of NG2^+^ cells (Fig. 5e). Altogether, these data indicate that Sirt1 plays a crucial functional role in HX-induced OPC proliferation.

### Stimulation of Cyc E/Cdk and Cdk2/Rb complex formation by HX

We have previously demonstrated that the Cdk2 signalling pathway plays a crucial role in regulating HX-induced OPC proliferation^14^. HX upregulated Cdk2, Cyc E and pRb (807/811) expression in white matter OPCs^14^. We first investigated whether HX regulates formation of specific Cdk/cyclin complexes. Immunoprecipitation of Cdk2 from white matter tissue followed by western blot for its specific regulatory subunit Cyc E confirmed enhanced formation of the Cdk2/Cyc E complex by HX, that is, activation of this pathway (Supplementary Fig. 8a–c). Previous data demonstrated that Cyc E is a positive regulator for both Cdk2 and Cdk1 (ref. 40), therefore we also investigated Cdk1/Cyc E interaction, and we observed HX-induced formation of the Cdk1/Cyc E complex (Supplementary Fig. 8b,c). Conversely, Cdk4/Cyc D and Cdk1/Cyc B1 complexes were not significantly altered by HX (Supplementary Fig. 8b,c). Previous studies demonstrated that Cdk1 can functionally compensate for the loss of Cdk2 by forming a complex with Cyc E (ref. 40). Our data indicate that Cdk1 can form a complex with Cyc E even in the presence of Cdk2, suggesting that Cdk1 may play a supportive role in HX-induced OPC proliferation.

**Figure 6:**
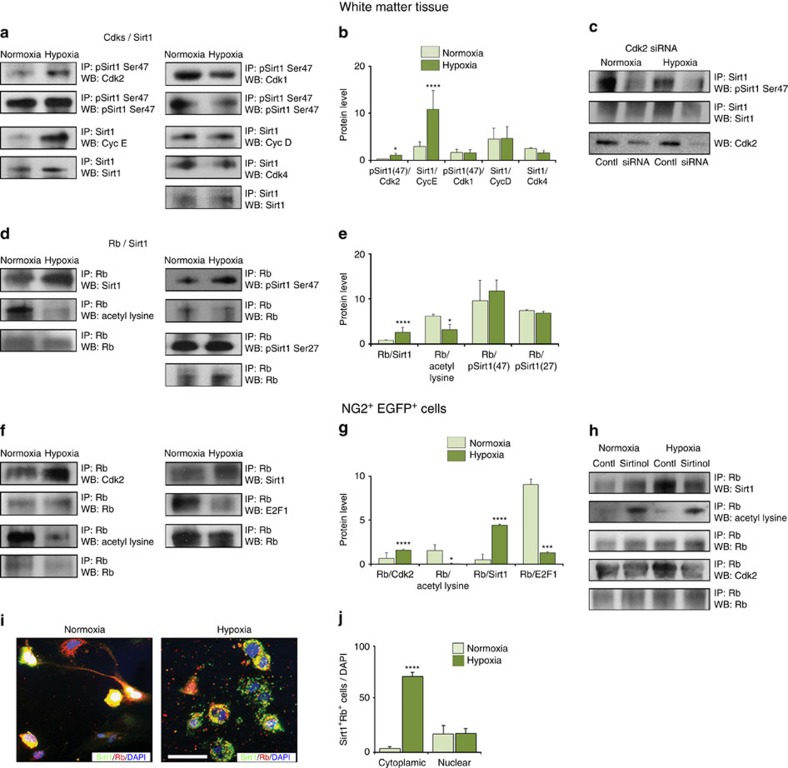
Neonatal HX promotes interactions between Sirt1 and specific components of the Cdk2 pathway. Representative western blots of Cdks/Sirt1 (**a**) and Rb/Sirt1 (**d**) complexes in NX and HX white matter (P18). Increased pSirt1 Ser47/Cdk2, Sirt1/Cyc E (**b**) and Rb/Sirt1 (**e**), and lower Rb/acetyl lysine (**e**) expression after HX. Histograms show mean±s.e.m.(*n*=3 brains per condition). (**c**) IP analysis in NX and HX cells transfected with Cdk2 siRNA shows less phosphorylated Sirt1 (*n*=3 brains per condition). (**f**) Representative western blot of FACS-purified NG2^+^ cells from CNP-EGFP mice (P18). (**g**) Western blot showing HX enhanced formation of Rb/Cdk2 and Rb/Sirt1, and reduced Rb/acetyl lysine and Rb/E2F1 (*n*=9 brains for each condition). (**h**) Representative western blot showing sirtinol treatment enhances Sirt1-mediated Rb acetylation in NX. In HX, deacetylation is reduced and Rb/Cdk2 expression. (**i**) Images of NX and HX cells stained with anti-Sirt1 and anti-Rb antibodies and DAPI. Scale bar, 50 μm. (**j**) Graph represents higher percentage of Sirt1^+^Rb^+^ cells in cytoplasm after HX. Histograms show mean±s.e.m. (three NX and four HX brains).

We then analysed formation of Cdk/tumour suppressor Rb complexes, as Cdks regulate Rb activity through its phosphorylation^41^,^42^. Immunoprecipitation (IP) analysis demonstrated interaction between Rb, and either Cdk1 or Cdk2 under NX conditions (Supplementary Fig. 8d,e). However, only Rb/Cdk2 complex formation was enhanced after HX, leading to Rb phosphorylation, as demonstrated by enhanced Rb807/811 phosphorylation (Fig. 6d,e). These findings indicate that Rb is a main substrate for HX-induced, Cdk2-mediated phosphorylation. In addition, we observed that HX did not affect Rb/Cdk1 complex formation (Supplementary Fig. 8d,e), indicating that Cdk2 is the main kinase that interacts with, and likely phosphorylates Rb after HX.

It has been shown that Rb forms complexes with E2F1 and E2F2 transcription factors^42^, and hyper-phosphorylation of Rb by Cdks causes dissociation of Rb/E2F complexes, thereby facilitating E2F-dependent transcription of genes that mediate S phase entry^43^. Therefore, we analysed regulation of Rb/E2Fs complex formation by HX. Higher levels of E2F1 and E2F2 were detected in white matter after HX, together with lower expression of the Rb/E2F1 complex (Supplementary Fig. 8f,g), indicating higher levels of unbound E2F1 in white matter after HX. No changes were observed in Rb/E2F2 complex levels (Supplementary Fig. 8f,g). These data indicate that elevated levels of unbound E2F1 are likely responsible for higher cell proliferation observed in white matter after HX.

Altogether, our data indicate that HX induces formation of the Cdk2/Cyc E, Cdk1/Cyc E, Rb/Cdk2 and pRb807/811/Cdk2 complexes and induces dissociation of the Rb/E2F1 complex, strongly suggesting that these proteins are functionally involved in HX-induced cell proliferation in white matter.

### Sirt1 regulates the Cdk2 pathway in white matter OPCs

To investigate whether Sirt1 regulates the cell cycle protein complexes identified above, we analysed the interaction between Sirt1 and these complexes by first determining their phosphorylation and acetylation status.

We first hypothesized that Cdk2 and Cyc E interact with Sirt1. Immunoprecipitation and western blot analysis of white matter tissue demonstrated that HX selectively enhanced formation of the pSirt1 Ser47/Cdk2 and Sirt1/Cyc E complexes (Fig. 6a,b). A recent report demonstrates that Sirt1 is mainly phosphorylated by Cdk1 (ref. 28), and we observed pSirt1 Ser47/Cdk1, Sirt1/Cyc D and Sirt1/Cdk4 complex formation in NX, but this was not modified by HX (Fig. 6a,b).

Since phosphorylated Sirt1 is found to be complexed with Cdk2, we determined whether the loss of Cdk2 would affect Sirt1 phosphorylation. Immunoprecipitation revealed that suppressed Cdk2 expression with Cdk2 siRNA in NX and HX white matter cells caused a decrease of pSirt1 Ser47 levels in cultured NX and HX white matter cells (Fig. 6c), indicating that Cdk2 regulates Sirt1 phosphorylation.

Since Rb activity is regulated by phosphorylation and acetylation^29^, we analysed Rb/Sirt1 complex formation and found that this was upregulated after HX (Fig. 6d,e). Furthermore, Rb deacetylation was also enhanced after HX (Fig. 6d,e), suggesting direct involvement of Sirt1 in Rb deacetylation. However, complex formation between pSirt1 Ser47 or pSirt1 Ser27 with Rb was not significantly altered with HX (Fig. 6d,e), suggesting that these phosphorylation events on Sirt1 are not specifically associated with Rb deacetylation in the context of HX.

Interactions between Sirt1 and Rb or Cdk2 induced by HX were also observed in FACS-purified NG2^+^ OPCs (Fig. 6f,g), indicating that all three proteins, Sirt1, Cdk2 and Rb, interact with each other during OPC proliferation induced by HX.

To study whether inhibition of Sirt1 activity altered Rb deacetylation after HX, we performed IP on NX and HX control cells and cells treated with sirtinol. Compared with NX cultures, HX enhanced the expression of Rb/Sirt1 complex, and Rb deacetylation was elevated (Fig. 6h). However, after sirtinol treatment, expression of the Rb/Sirt1 complex was reduced after HX, with increased expression of acetylated Rb, indicating that deacetylation of Rb is mostly dependent on Sirt1 activity. Also, after blocking Sirt1 activity through sirtinol, HX reduced the expression of the Rb/Cdk2 complex (Fig. 6h), suggesting that Sirt1 plays an important role in this complex formation. Finally, since we demonstrate a strong interaction between Sirt1 and Rb after HX, we additionally investigated a subcellular co-localization of these proteins in NX and HX cultures. *In vitro* immunolabelling revealed that under NX conditions Sirt1 co-localized with Rb mainly in nucleus; however, HX altered Sirt1 and Rb co-localization into cytoplasm (Fig. 6i,j). This suggests an interactive role of Sirt1 and Rb in cytoplasm under HX conditions.

### Neonatal HX induces Sirt1 translocation to the cell cytoplasm

Sirt1 deacetylates both histone and non-histone proteins, including a number of transcription factors^27^. Thus, changes in subcellular localization are likely to play an important role in the regulation of many Sirt1-dependent cellular processes^44^. Interestingly, a nucleo-cytoplasmic shuttling of Sirt1 on oxidative stress has been previously demonstrated^45^,^46^.

We observed that under NX *in vivo*, Sirt1 was mainly localized in the nucleus of white matter cells, although some cytoplasmic immunostaining was also observed (Supplementary Fig. 9a). However, the intensity of Sirt1 nuclear staining decreased by ∼80% over a 120 min time period after HX. A reduction of ∼60% was observed within the first 15 mins after HX, followed by a further time-dependent decline (Supplementary Fig. 9b). After HX, a significant subpopulation of white matter cells displaying Sirt1 expression exclusively in the cytoplasm was observed (Supplementary Fig. 9c). In both NX and HX white matter, a small percentage of cells displayed expression of Sirt1 in both nucleus and cytoplasm (Supplementary Fig. 9c).

The subcellular distribution of Sirt1 was further investigated and quantified by western blot analysis, using nuclear and cytoplasmic fractions obtained from NX and HX white matter tissue. Sirt1 was mainly detected in the nuclear fraction under NX, and Sirt1 nuclear levels were reduced after HX (Supplementary Fig. 9d,e). Conversely, higher Sirt1 levels were detected in the cytoplasmic fraction after HX, as confirmed using specific anti-MEK 1/2 antibody (Supplementary Fig. 9f). Furthermore, analysis of subcellular localization of Sirt1 revealed high levels in the mitochondrial compartment, but no co-localization with anti-Golgin-97 antibody in Golgi apparatus (Supplementary Fig. 9f). Since Sirt1 is synthesized in the cytoplasm, these data indicate that either HX prevents Sirt1 shuttling to the nucleus, or causes re-shuttling from the nucleus to the cytoplasm. In either case, increased Sirt1 in the cytoplasm likely has important functional consequences, as this subcellular localization could facilitate its interaction with cytoplasmic shuttling of Rb protein^47^.

### Sirt1 inactivation decreases Cdk2 and Rb complex formation

**Figure 7:**
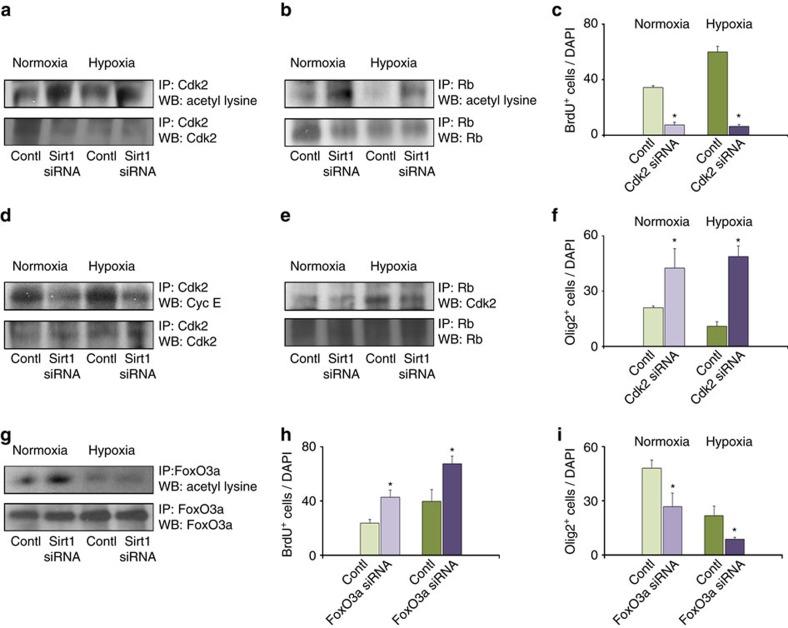
Knockdown of Sirt1 reduces Cdk2/Cyc E and Cdk2/Rb complex formation. Representative western blots showing levels of acetylated Cdk2 (**a**), acetylated Rb (**b**), Cdk2/Cyc E (**d**) and Rb/Cdk2 complexes (**e**), and acetylated FoxO3a (**g**) in NX and HX white matter (P18), in the presence or absence of Sirt1 (*n*=3 brains for each group, for each treatment). Graphs represent percentages of proliferating BrdU^+^ cells in control cultures and after knockdown of Cdk2 (**c**) or FoxO3a (**h**) and Olig2^+^ cells after knockdown of Cdk2 (**f**) or FoxO3a (**i**) in NX and HX cultures. Knockdown of Cdk2 reduces the percentage of proliferating cells and elevates Olig2^+^ cells in NX and HX cultures as compared with control cultures. However, knockdown of FoxO3a increases BrdU^+^ cells and reduces Olig2^+^ cells in NX and HX cultures. Histograms show mean±s.e.m. Three cultures per condition.

To determine the role of Sirt1 in modulating the ability of Cdk2 to form complexes with Cyc E and Rb, we performed IP analysis on NX and HX cells from white matter transfected with Sirt1 siRNA or scrambled control. Under these conditions, a reduction in Cdk2 and Rb deacetylation was observed, along with a reduction in Cdk2/Cyc E and Cdk2/Rb complex formation. (Fig. 7a,b,d,e). These data indicate that loss of deacetylation by Sirt1 decreases Cdk2/Cyc E and Cdk2/Rb complex formation, which likely decreases cell proliferation. Consistent with this notion, proliferation assays demonstrated a significant reduction in the percentage of BrdU^+^ cells in both NX and HX cultures treated with Cdk2 siRNA. This was accompanied by an increased percentage of cells expressing Olig2^+^ (Fig. 7c,f), consistent with the interpretation that Cdk2 activity, similar to that of Sirt1, supports basal and HX-induced progenitor cell proliferation at the expense of differentiation and Olig2^+^ cell formation.

It has been previously demonstrated that Sirt1 deacetylates and represses the activity of FoxO transcription factors, including FoxO1, FoxO3a and FoxO4 (refs 48, 49). In NX and HX white matter cells, FoxO3a acetylation is increased in the absence of Sirt1 (Fig. 7g). Knockdown of *FoxO3a* gene in both NX and HX cells enhanced their proliferation and reduced the percentage of Olig2^+^ cells (Fig. 7g–i). These results point to an interaction between Sirt1 and FoxO3a, and opposing roles for Sirt1 and FoxO3a in OPC proliferation and OL differentiation.

**Figure 8:**
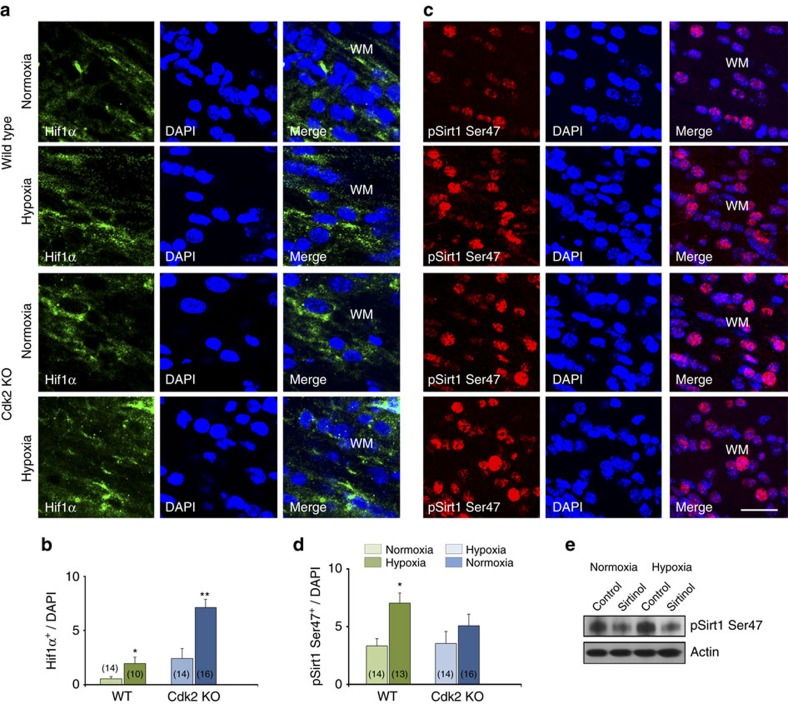
Sirt1 phosphorylation by Cdk2 requires sirtuin's deacetylase activity. Confocal images from NX and HX white matter from WT and *Cdk2* KO mice. Cells were immunolabelled with anti-Hif1α (**a**) and anti-pSirt1 Ser47 (**c**) antibodies. Graphs demonstrate percentages of Hif1α (**b**) and pSirt1 Ser47 (**d**) cells in HX white matter in both genotypes. Histograms show mean+s.e.m. Number in parentheses within bar indicates number of samples; four NX and six HX brains. (**e**) Representative western blot demonstrates lower expression of pSirt1 Ser47 in NX and HX conditions after sirtinol treatment. Actin was used as a loading control (*n*=3 brains for each condition, for each genotype).

To investigate the causal relationship between Cdk2 and Sirt1 activity, we used Cdk2 KO mice, in which the *Cdk2* gene was silenced in exons 4 and 5 (ref. 50). We immunolabelled white matter tissue with anti-pSirt1 Ser47 and anti-Hif1α antibodies. In both *WT* and *Cdk2* KO littermates, HX selectively increased the percentage of Hif1α^+^ cells in white matter (Fig. 8a,b). In contrast, *Cdk2* KO abolished the phosphorylated Sirt1 cellular response to HX (Fig. 8c,d), indicating that HX-induced phosphorylation of Sirt1 at Ser47 is dependent on Cdk2. This indicates that Cdk2 does not positively regulate Hif1α as an upstream factor, consistent with the findings of Hubbi *et al*.^51^, but is required for Sirt1 phosphorylation. To determine whether a hierarchy exists between Cdk2 deacetylation and Sirt1 phosphorylation, we tested the possibility that Sirt1 deacetylase activity was required for Sirt1 phosphorylation. Inhibition of Sirt1 activity with sirtinol resulted in decreased pSirt1 Ser47 expression in both NX and HX conditions (Fig. 8e). Taken together, these data demonstrate that *Cdk2* deletion does not decrease Hif1α expression induced by HX, and strongly suggests that the phosphorylation of Sirt1 at Ser47 by Cdk2 requires Sirt1 deacetylase activity.

Altogether our molecular data indicate that HX increases levels of Hif1α in parallel with NAD, which, respectively, elevates Sirt1 expression and induces its activation. Increased levels of activated Sirt1 in turn deacetylate Cdk2 and Rb. Deacetylation of Cdk2 leads to phosphorylation of Sirt1 and Rb. Rb deacetylation and phosphorylation facilitate dissociation of the Rb/E2F1 complex. Unbound E2F1 is crucial for G1/S transition, and its release from the Rb complex results in enhanced OPC proliferation after HX (Supplementary Fig. 10).

## Discussion

In the present study, we demonstrate that the epigenetic modulator Sirt1 is functionally involved in the expansion of the endogenous OPC pool in the NX-developing brain and after neonatal HX. We show that OPC proliferation in both conditions is controlled by the Cdk2 signalling pathway, in particular by post-translational modifications of Cdk2 and Rb that depend on Sirt1 activation. We also demonstrate that HIF1α activation occurs after HX with a time course consistent with a role in OPC proliferation and increased Sirt1 activity. Also, a higher level of NAD increases Sirt1 activity as indicated by a lower level of Cdk2 acetylation. Finally, we demonstrate that inhibition of Sirt1 expression *in vitro* and *in vivo* not only prevents OPC proliferation but also promotes OL maturation in HX white matter. These findings reveal that optimal Sirt1 activity is essential to balance OPC proliferation and OL regeneration.

In the present study, Sirt1 expression is specifically upregulated in proliferating white matter NG2^+^ OPCs. Our gain- and loss-of-function studies indicate that Sirt1 is a part of basic regulatory mechanism of OPC proliferation, reducing cell proliferation after Sirt1 siRNA knockdown and increasing proliferation after Sirt1 overexpression. In contrast to earlier observations in the adult brain^52^, which reported that Sirt1 is absent in mature astrocytes, we detected its expression in a small percentage of GFAP^+^ and Iba1^+^ cells of the developing white matter. Importantly, as Sirt1 shows both Hif1α-dependent and Hif1α-independent functions in proliferation, these studies establish Sirt1 as an essential component of the regulatory mechanism that controls OPC proliferation in NX, which is also clearly recruited in the cellular response to HX.

After chronic neonatal HX, increased cytoplasmic Sirt1 in OPCs strongly correlates with their proliferative state, suggesting that Sirt1 is a main regulator of the proliferative response of OPCs. Direct functional evidence was obtained both *in vitro* and *in vivo*, demonstrating that downregulation of Sirt1 protein expression in cultured OPCs, as well as excision of the *Sirt1* gene in *Sirt1-*cKO mice severely reduced OPC expansion after HX, indicating that *Sirt1* inactivation reduces the OPC response to neonatal white matter injury.

Our previous studies on rodent model of chronic HX, which recapitulates many cellular and functional characteristics in focal ischaemic injuries in preterm infants, demonstrated that p27^Kip1^, main Cdk2 inhibitor, is involved in OL differentiation in hypoxic white matter^14^. Since Sirt1 negatively regulates p27^Kip1^ expression^53^, the increased Sirt1 that is observed in this model is consistent with decreased p27^Kip1^ levels after white matter injury. Previous analysis of the adult white matter demonstrated that genetic inactivation of *Sirt1* expanded the OPC pool after demyelination, that is, that Sirt1 inhibited demyelination-induced OPC proliferation in the adult brain^52^. The opposite regulation of Sirt1 in proliferating OPCs in neonatal versus adult white matter injury could be related to intrinsic differences in OPC properties and/or changes in the cellular environment that occur during white matter maturation. Differences in the type of injury used in these experimental models (global hypomyelination induced by HX versus focal demyelination) also affect Sirt1 regulation. Consistent with Rafalski's studies^52^ and opposite to HX, our analysis of Sirt1 response after lysolecithin-induced demyelination demonstrated a downregulation of Sirt1 expression in adult NG2^+^ progenitors, which may indicate a context-dependent role for Sirt1 function in the regenerative response of OPCs.

Our previous studies demonstrated that under pathological conditions NG2^+^ cells generate OLs^35^. Here we showed that NG2^+^, not Mash1^+^, progenitor cells are particularly susceptible to changes in Sirt1 expression after HX. HX-specific response of NG2^+^ progenitors expressing Sirt1 can significantly inhibit the maturation of OLs, which are necessary for myelin formation. It is possible that Sirt1 upregulation serves to replenish the OPC pool as these regenerate OLs, and other signals may later initiate the decline of Sirt1 activity to allow OPC differentiation and white matter recovery. When we investigated the regional specificity of Sirt1 function after HX, we found that—differently from white matter—inactivation of *Sirt1* had no effect on proliferation of neural progenitor cells in the neonatal SVZ, where we still detected a higher number of proliferating Ki67^+^ cells and NG2^+^ progenitors. These results are in agreement with analysis of the adult SVZ, demonstrating that inactivation of *Sirt1* enhances neural stem cells (NSC) proliferation^52^. Taken together, the analyses of the developing and adult brain discussed above supports the notion that Sirt1 plays a crucial temporally and spatially restricted role in glial regeneration after brain injury.

In the adult brain, deregulation of the sirtuin pathways has been implicated in the pathogenesis of various neurodegenerative disorders, including Alzheimer's, Parkinson's and Huntington's disease, and in multiple sclerosis^17^,^54^. In these pathologies, Sirt1 plays a neuroprotective role through activation of multiple downstream targets^55^,^56^. Sirt1 activation has also been established in HX/ischaemia and in oxidative stress^57^. Sirt1 responsiveness to oxygen concentrations is regulated by the HX-induced factors HIFs, specifically the HIF1α and HIF-2α isoforms^23^. Our results in the developing brain demonstrate that chronic HX enhanced HIF1α expression, and that HX-induced Sirt1 expression was HIF1α-dependent. In summary, our data point to HIF1α-dependent Sirt1 activation as a major regulatory step in white matter OPC proliferation. Interestingly, *in vivo* analysis of HIF1/2 KO mice revealed reduced OPC proliferation at E18 (ref. 37), suggesting that a similar regulatory pathway could also exist in development.

A number of studies demonstrated that small molecules either activating or inhibiting Sirt1 mitigate the cellular consequences of many neurological disorders and cancer^17^,^58^. Our *in vitro* data demonstrate that inhibition of Sirt1 activity either by siRNA or by the Sirt1 inhibitor sirtinol promotes oligodendrogenesis after HX. Although therapeutic use of this inhibitor is limited by its low potency and the lack of high specificity, the effectiveness of Sirtinol to induce cell growth arrest has already been demonstrated in cancer cells, pointing to potential clinical applications^39^. In our chronic HX model, sirtinol treatment accelerated OL regeneration and maturation, which represent crucial steps in white matter regeneration after neonatal injury^16^. Therefore, modulation of Sirt1 activity by sirtinol might also hold potential therapeutic implications for DWMI in prematurely born infants.

In agreement with the notion that Sirt1 activity is regulated by nucleo-cytoplasmic shuttling and post-translational modifications in Sirt1 alter its subcellular localization^46^, we found significant changes in the subcellular localization of Sirt1 in white matter cells after neonatal HX. Extrinsic factors can alter subcellular localization of Sirt1 (ref. 27), for example, cell culture conditions promoting neuronal differentiation induce cytoplasmic–nuclear translocation of Sirt1 (ref. 44). We found that under NX conditions, Sirt1 was mostly localized in the nucleus, but HX promoted subcellular translocation to the cytoplasm, where Sirt1 was found additionally to be localized in the mitochondrial compartment. These findings suggest that nucleo-cytoplasmic shuttling of Sirt1 and Sirt1–Rb co-localization play an important role in HX-induced OPC proliferation. Since the predominantly nuclear Sirt1 also regulates basal proliferation, it is possible that HX-induced Sirt1 translocation need not be restricted to proliferation, and suggests inclusion of metabolic targets, including mitofusin-2, whose deacetylation regulates mitochondrial function and cell death after ischaemic/reperfusion injury^59^.

Our present study provides evidence for a novel regulatory mechanism of OPC proliferation under normal and pathological conditions, demonstrating that Sirt1 regulates both Cdk2 signalling and Rb activity. Neonatal HX activates Cdk2, which is essential for OPC proliferation in white matter after neonatal injury^14^. Our analysis indicates that neonatal HX enhances Sirt1 activation in OPCs, which in turn promotes Cdk2 and Rb deacetylation, and dissociation of the Rb/E2F1 complex. Release of E2F1 from the Rb/E2F1 complex plays a crucial role in maintaining OPC proliferation and preventing cell cycle exit. Activation of the Cdk2/Rb/E2F1 pathway has been reported in other animal models of pathology, in particular in frontal cortex neurons after repeated electroconvulsive shock and in cerebellar granule neurons after kainic acid treatment^60^. However, these studies demonstrated activation of the Cdk2/Rb/E2F1 pathway in neurons, but they did not establish a correlation with an enhanced neuronal cell proliferation.

In our model of chronic neonatal HX, we show that different Cdk/cyclin complexes are activated after injury and therefore could be involved in post-HX OPC expansion. We demonstrate formation of both Cdk2/Cyc E and Cdk1/Cyc E complexes induced by neonatal HX. Cdk1 is the major binding partner of Cyc B1, which regulates M phase checkpoint of the cell cycle^61^, but Cdk1 could also function as the catalytic partner of Cyc E, particularly in the absence of Cdk2 (ref. 40). However, we found that in the presence of Cdk2, both Cdk2/Cyc E and Cdk1/Cyc E complexes are expressed, indicating that both kinases bind to Cyc E in the normal developing white matter.

Our results show that post-translational changes in individual members of the Cdk2 signalling pathway are regulated by Sirt1 activity under HX conditions. Previous reports demonstrated formation of a Sirt1/Cdk1 complex, and subsequent phosphorylation of Sirt1 by Cdk1 kinase, whereas other G1-associated kinases were not detected to interact with Sirt1 (ref. 27). Our analysis strongly indicates that Sirt1 is a previously uncharacterized substrate of Cdk2-mediated phosphorylation, as shown by the loss of pSirt1 Ser47 after Cdk2 knockdown and ablation. Similar to our data, it has been shown that Cdk5 also phosphorylates Sirt1 at Serine 47 (ref. 62) Sirt1 phosphorylation has been implicated in its stability^28^ and translocation^62^, but its multiple kinases, including Jun-N-terminal kinase^63^ and mTOR^64^, suggest this site to be related to substrate-specific functions in different contexts^65^. Furthermore, our finding that Sirt1 phosphorylation is abolished by sirtinol suggests the presence of feed-forward regulation of Sirt1 via Cdk2 that is itself dependent on Sirt1 deacetylase activity; this may provide modulation of Sirt1 output according to target demand.

Our immunoprecipitation analysis suggests that both Cdk1 and Cdk2 might be involved in cell cycle progression in white matter OPCs; however, only Cdk2 is deacetylated by Sirt1 in HX. In the present study, we show that HX-induced NAD increase activates Sirt1 as revealed by higher Cdk2 deacetylation. Hif1α elevated in HX upregulates expression of Sirt1, which is upstream of Cdk2 and subsequently deacetylates Cdk2 in OPCs through formation of a Sirt1/Cdk2 complex. We also show that Sirt1 interacts with Rb, which is deacetylated after HX. These results are consistent with previous data demonstrating NAD-dependent, Sirt-1-mediated Rb deacetylation in cultured cell lines^29^.

On the basis of our findings that higher level of unbound E2F1 could be detected in white matter progenitors after HX, we propose a molecular mechanism underlying OPC proliferation and pool expansion induced by neonatal brain injury (Supplementary Fig. 10). Elevated Sirt1 activity induced by HX and higher levels of NAD promotes Sirt1/Cdk2 interaction. This causes deacetylation of Cdk2 by Sirt1 and of its phosphorylated target, Rb. Deacetylation of Rb by Sirt1—as well as hyper-phosphorylation of Rb by Cdk2—is required for dissociation of the Rb/E2F1 complex and release of the transcription factor E2F1, which promotes entry into S phase of the cell cycle. Sirt1 is in turn phosphorylated by Cdk2, which may further regulate its activity. Altogether, this sequence of events results in an elevated number of proliferating OPCs in white matter after HX. Our observation of Sirt1/Rb co-localization is consistent with Sirt1 and Rb nucleo-cytoplasmic shuttling^44^,^47^.

Our study demonstrates a crucial functional role for the HDAC Sirt1 in maintaining proliferation of the endogenous pool of OPCs in the developing white matter. OPCs represent the major and most abundant cell population involved in OL regeneration and white matter recovery after injury^14^,^66^,^67^. We also define the molecular mechanisms by which HX-induced elevation of NAD stimulates Sirt1 deacetylase activity to promote injury-induced OPC proliferation. In particular, we identify Sirt1 targets within the Cdk2/Rb/E2F1 pathway. The finding that gain- and loss-of-function of Sirt1—as well as its pharmacological inhibition—alters OPC proliferation and their differentiation to mature OLs indicates that Sirt1 may serve as a potential target for therapeutic interventions promoting OL regeneration after HX-induced neonatal brain injury.

## Methods

### Animals

The colonies of WT (C_57_BL/6, catalogue #003548, Jackson Laboratory), CD1 (Crl:CD1(ICR), Charles River), *CNP-EGFP* (generated by Dr V. Gallo, Children's National Medical Center, Washington, DC), *Sirt1*^*F/F*^*;PDGFRαcreER*^*T2*^*;Rosa*^*YFP*^–*Sirt1*-cKO, *HIF1α* KO mice (catalogue #008041 and #024640, respectively, Jackson Laboratory) and *Cdk2* KO mice were maintained in the animal facility of the Children's National Health System according to the Institutional Animal Care and Use Committee and the National Institutes of Health guidelines. The *VHLfl/fl* mice^68^ and *PlpCreERT2* (ref. 69) mice were maintained in the UCSF animal facility according to the Institutional Animal Care and Use Committee and the National Institutes of Health guidelines. To change the genetic background, heterozygote *CNP-EGFP*^+^ males were backcrossed to C_57_BL/6 females for more than eight generations. To obtain mice in which the *Sirt1* gene could be conditionally excised in OPCs, we crossed Sirt1^F/F^ mice (where lox sites flanked exon 4 of the *Sirt1* gene) with *PDGFRαcreER*^T2^ transgenic mice (*PDGFRαcreER*^T2^), which express the tamoxifen-inducible form of *Cre* in OPCs expressing *PDGFRα*. To trace OPCs with excised *Sirt1*, we crossed Sirt1^F/F^;*PDGFRαcreER*^T2^ with mice carrying a fluorescent reporter gene, enhanced yellow fluorescent protein (eYFP). Cre/lox recombination leads to the deletion of STOP sequence and expression of eYFP. Therefore, tamoxifen injection induced both *Sirt1* ablation and permanent eYFP expression in OPCs. After birth, *Sirt1-*cKO mice were genotyped by PCR according to Jackson Laboratory protocol. DNA products were loaded onto 2% agarose gels to resolve the specific bands.

### Hypoxia paradigm

All mice (3 days old, P3) were exposed to 9.5–10.5% oxygen concentration in a HX chamber. To maintain HX conditions, nitrogen was added to displace oxygen. Oxygen concentration was maintained and monitored continuously with the sensor inside the chamber. To optimize nutrition during HX, transgenic pups were housed in the chamber with two CD1 foster mothers and their pups. At P11, mice were removed from the chamber and transferred to a room with normoxic air conditions, remaining under the foster mothers' care to minimize stress. For all strains, exposure to HX lasted 8 consecutive days (P3–P11). After removal from the HX chamber, mice were subjected all experimental conditions at the specified time points. To abolish *Sirt1* expression in OPCs, NX and HX *Sirt1-*cKO mice were injected with tamoxifen intraperitoneally twice—at P12 and P13 (50 mg per g of body weight each)—and then killed at P18.

### Lysophosphatidylcholine-induced demyelination

Demyelination was performed in adult (P40–P60) WT mice after deep ketamine/xylazine anaesthesia (10 mg per g body weight). Mice were placed in a stereotaxic frame (Stoerling) and injected into the CC with 2 μl of 1% lysophosphatidylcholine solution and/or 0.9% NaCl using Hamilton micropipettes (Stoerling). Injection time lasted 5 min to reduce reflux along the needle track. Stereotaxic coordinates for corpus callosum (CC) were taken from bregma: 0.26 mm caudal; 1.0 mm lateral; and 2.5 mm ventral. The day of injection was designated as day 0. Mice were processed for immunocytochemistry after lysophosphatidylcholine or control NaCl injections.

### Immunocytochemistry and cell counting

Immunocytochemistry was performed on floating sections using antibodies^14^ against the following antigens: NG2 (1:500; Chemicon, AB5320); GFAP (1:1,000; Abcam ab4674-50); BrdU (1:200; Becton Dickinson, 347580); Ki67 (1:250; NovoCastra, NCL-Ki67p); Olig2 (1:500; Abcam, ab33437); Mash1 (1:400; BD Bioscience, 556604); CC1 (1:250; CalBiochem, OP80); Sirt1 (1:250; Cell Signaling, 2028); Iba1 (1:500; WAKO, 019-19741); and CD38 (1:500; AbD Serotec MCA1957). Sections were incubated overnight at 4 °C in primary antibodies diluted in 0.1 M PBS (pH 7.4), containing 0.1% Triton and 5% normal goat serum. Appropriate secondary antibodies were used as follows: TRITC-conjugated AffiniPure Goat Anti-Mouse IgG (H+L); FITC-conjugated AffiniPure Goat Anti-Rabbit IgG; and TRITC-conjugated AffiniPure Goat Anti-Mouse IgM (115-025-146, 111-095-008 and 115-025-020, respectively, all from Jackson ImmunoResearch). Sections were incubated with secondary antibodies for 1 h at room temperature and then mounted. *Z*-stacks of 1 μm-thick optical sections through the entire thickness of slice were captured using a confocal microscope (MRC1024, BioRad Laboratories) and collapsed along *z* axis before cell counting. Measurements were taken from 7–12 tissue sections obtained from 3–4 mice in each group. The results are presented as a mean±s.e.m. and *t*-test was performed to test the hypothesis of means' equality.

### Cell cultures

White matter was dissected from 300 μm-thick brain sections obtained from NX and HX mice at P18, and digested in Hanks' balanced salt solution (Gibco, 14170-161) containing papain (13 units per ml, Sigma, T4762), DNAse (5 units per ml, Sigma, D5427) and trypsin (Sigma, T4799) for 30 min at 37 °C. Cells were dissociated by trituration and resuspended in Hanks' buffer containing 1 M HEPES (BioSource, P305), 15% sucrose and penicilin/streptomycin. Cells were then plated onto poly-L-lysine-coated dishes at a density of 10 cells per μl, and cultured for 10 days in D-MEM/F-12 medium (Gibco, 11330-032), supplemented with 1% N2 and 1% B27 (Gibco, 17502-048, 17504-044), and with 20 ng ml^−1^ epidermal growth factor (EGF) and 10 ng ml^−1^ basic fibroblast growth factor (bFGF; Upstate Biotechnology, 01–407, 01–114). To immunolabel differentiated cells, standard protocols were used^14^ with primary antibodies against GalC (Galactocerebroside), O4 and Olig2 (AB142, MAB345 and AB9610, respectively, all from Chemicon).

To study Sirt1 activity, NX and HX cells from white matter were treated with nicotinic acid riboside (Toronto Research Chemicals, N429400) or β-nicotinamide adenine dinucleotide (Fisher Scientific, ICN10116801) for 24 h and then washed out in control medium

To inhibit Sirt1 activity, 100 μm sirtinol (Calbiochem, 566321) has been added to medium for 24 h. Then NX and HX cells were washed out of sirtinol and cultured in medium with 20 ng ml^−1^ EGF and 10 ng ml^−1^ bFGF (Upstate Biotechnology, 01–407, 01–114) for 8 days. For immunoprecipitation, cells were lysed in RIPA buffer with protein inhibitors and then processed accordingly.

### OPC cultures

Mouse OPCs were isolated and purified by immunopanning from P7 mouse cerebral cortices, and then plated and maintained using previously described methods^37^. Briefly, mouse cerebral cortical tissue underwent enzymatic dissociation, and OPCs were isolated by depletion of microglia with BSL1, followed by immunopanning for PDGFRa. Cells were maintained in proliferation conditions with the addition of CNTF, PDGF and NT-3 (Peprotech) to base medium. Tamoxifen (Sigma) was added to cultures at 24 h after plating and replenished every other day in medium. OPCs were then collected in Trizol (Life Technologies). RNA was isolated using phenol–chloroform purification followed by the RNeasy mini kit with RNase-free DNase kit (Qiagen). cDNA was made using a High Capacity cDNA kit according to the manufacturer's instructions (Applied Biosystems), and RT–PCR was performed. Samples were then run on a 2% agarose gel. Sirt1 bands were quantified using ImageJ for density and normalized to GAPDH density.

### Fluroescence-activated cell sorting

White matter was dissected from NX and HX mice at P18. Isolated cell suspensions were FACS-purified as previously described^14^ (Influx, Cytopeia, Seattle, WA). Briefly, tissue was dissocated in Hanks' balanced salt solution containing 0.1% trypsin and 100 U ml^−1^ DNase 1 (Sigma, St Louis, MO) for 20 min. Following incubation with 0.7 mg ml^−1^ trypsin inhibitor (Sigma) and trituration, cell suspensions were analysed for light forward and side scatter using a FACStar Plus instrument (Becton Dickinson, Franklin Lakes, NJ). After collection and washing with D-MEM/F12 medium, purified CNP-EGFP^+^ cells were plated on 24-well dishes coated with poly-lysine at the density of 10 cells per μl, and cultured in SCM (Stem Cell Technology, Inc.) containing 20 ng ml^−1^ EGF and 10 ng ml^−1^ bFGF (Upstate Biotechnology). To isolate NG2^+^ progenitors, cell suspensions obtained from NX and HX CNP-EGFP mice were incubated with anti-NG2 antibody (1:1,000; Chemicon) for 1 h at 4 °C. Cells were then washed twice in D-MEM/F12 medium and incubated with Alexa 647 (Jackson Immunolaboratory) for 1 h at 4 °C. NG2^+^ labelled cells were FACS-purified as previously described (Influx, Cytopeia)^14^ by incubation with NG2 primary antibodies and R-phycoerythrin-conjugated secondary antibodies.

### Cell transfection with siRNA

Cell transfections were performed using the NeuroPORTER Transfection reagent (Genlantis, T400750) according to the manufacturer's instructions. After white matter dissection, cells were plated in 12-well cell culture dishes at a density of 50 cells per μl for 24 h. At the time of transfection, cell cultures were ∼60% confluent. Commercially available siRNA directed against Sirt1 (Ambion, NM-009870), Cdk2 (Ambion, AM16704) and Foxo3a (Ambion, AM16708) used at 20 pM produced selective gene knockdown at 7 h post transfection. Briefly, 2 μl of 20 pM siRNA solution and 12 μl of the transfection reagent were incubated in 100 μl of OptiMEM medium (Gibco) for 20 min to facilitate complex formation. The siRNA transfection mix was added to the cells cultured in 10% FBS. The control consisted of nonspecific siRNA (Silencer Negative control No. 1, AM4635, Ambion). Cells were transfected for 7 h at 37 °C, washed with Hanks' buffer and cultured in MEM with 10% FBS for an additional 24 h. The medium was then changed to Stem Cell Medium (20 ng ml^−1^ EGF and 10 ng ml^−1^ FGF). To demonstrate Sirt1 knockdown, western blot analysis was performed on transfected NX and HX cells using anti-Sirt1 antibody. To assess the proliferative potential of cells, they were incubated in the presence of BrdU (10 μg ml^−1^) for 60 min at 37 °C. Cells were then fixed in 4% paraformaldehyde and kept in PBS until use. BrdU incorporation was visualized by immunofluorescence using anti-mouse BrdU antibody and TRITC-conjugated AffiniPure Goat Anti-Mouse IgG (H+L). Percentages of BrdU^+^ cells were quantified in random fields captured under × 10 magnification (total of more than 250 cells) from at least three different samples and subjected to statistical analysis.

### Western blot and immunoprecipitation

White matter samples from NX and HX mice were homogenized in RIPA lysis buffer with proteinase inhibitors (Santa Cruz Biotech, Inc., Sc 24948). Protein extracts were boiled for 5 min before loading onto 4–20% gradient gels (GeneMate, E4326-420, 20 μg of protein per each lane). Gels were electrotransfered to a 0.2 μm nitrocellulose membrane (Millipore). Blots were blocked in 5% milk in TBST for 1 h, and then incubated at 4 °C overnight with one of the following antibodies: anti-Cdk1; -Cdk2; -Cdk4; -Cyc D; -Cyc E; -Cyc B; -Rb; -pRb 807/811; -E2F1; -E2F2; Sirt1 (Santa Cruz Biotechnology, sc-54, sc-6248, sc-260, sc-25765, sc-48420, sc-166210, sc-50, sc-16670, sc-193, sc-633 and sc-15404); pSirt ser47 and pSirt ser27 (Abcam, ab76039 and ab76029); phospho-H3; acetylo-lysine (Cell Signaling 9681,9713); -actin (Chemicon, MAB 1501R); and -HIF1α antibody (Novus, NB100-123). Bands were detected with appropriate horseradish peroxide-conjugated secondary antibodies, reacted with chemiluminescent ECL substrate (Amersham, RPN2132) and visualized by exposure to X-ray-sensitive film. Band intensity was measured using the ImageJ programme (NIH). Full western blots are available as Supplementary Figs 11 and 12.

For immunoprecipitation, white matter tissue extracts were prepared from NX and HX mice at P18 in RIPA buffer containing 2%Triton X-100 and 0.2% SDS. Aliquots (270 μg tissue) were incubated overnight with antibodies against various antigens and 15 μl of agarose A (sc 2001, Santa Cruz Biotechnology). Complexes bound to agarose A were collected by centrifugation and washed twice in 500 μl of RIPA buffer containing proteinase inhibitors. Precipitated proteins were analysed by immunoblotting with antibodies against expected co-precipitating proteins. Bands were detected by using horseradish peroxide-labelled polyclonal anti-mouse or anti-rabbit antibodies (554002 and 554021, BD Biosciences) and developed with a chemiluminescent substrate (ECL, Amersham).

### Reverse transcriptase PCR

cDNA was first synthesized from 1.5–2 μg of total RNA extracted from NX and HX cultured cells in the total volume of 11 μl, including 10 mM dNTP mix and 0.5 μg μl^−1^ Oligo dT (Invitrogen, 12371-019). Reaction mixtures were heated at 65 °C for 5 min and then at 42 °C for 50 min. From 2 μl of cDNAs, sequences of interest were amplified in a thermocycler in a total volume of 25 μl of mixture with Tag Polymerase. Primer sequences are provided as Supplementary Information. PCR products were resolved by vertical electrophoresis on 2% agarose gels. Band intensity was measured using ImageJ programme (NIH). The sequences of primers were listed in Supplementary Table 1.

### NAD quantification

NAD assay was performed on white matter tissue dissected from NX and HX mice at P18 by using the NAD^+^/NADH Quantification Kit (BioVision K-337-100). A unit of 20 μg samples were taken for analysis, washed with cold PBS, homogenized with 400 μl of NAD^+^/NADH extraction buffer and spun down at 14,000 r.p.m. for 5 min. To detect NADH only, NAD^+^ was decomposed before the assay by heating samples to 60 °C for 30 min in a water bath, and then cooling on ice. Precipitates were removed by centrifugation, and NAD^+^-decomposed samples were transferred to 96-well plates (50 μl volume in duplicates). Both, total NAD^+^/NADH and NADH-only samples were incubated with detecting reagents. Standard curve dilutions were incubated with NAD^+^ Cycling Mix (NAD^+^ Cycling buffer—100 μl and NAD^+^ Cycling enzyme mix—2 μl), then 10 μl of NADH developer was added to each well, and samples were incubated for 2 h at room temperature. Plates were read at 450 nm wavelength. The reaction was stopped by adding 10 μl of stop solution to each well and mixing. The standard curve was generated and the amount of the nucleotides was calculated based on NAD^+^/NADH and NADH-only readings. The concentration of NAD^+^/NADH ratio or NADH was expressed as ng per mg protein or pmol/10^6^.

### HDAC fluorometric activity assay

HDAC activity assay was performed on normoxic and hypoxic tissue. After tissue digestion (20 μg) in RIPA buffer, 25 μl of HDAC Assay Buffer (Enzo, 100010996) was added to each well. The HDAC Assay Buffer contained 0.2 mM trichostatin to inhibit histones class I and II, or 50 mM nicotinamide to block sirtuins (class III). Then, extracts were incubated in Fluor-de-Lys substrate in room temperature. After 15 min, 0.2 mM trichostatin and 50 mM nicotinamide were added to each sample and samples were quantified in a microtitre-plate fluorimeter (excitation 350–380 nm and detection 440–460 nm).

### Statistical analysis

All animal experiments concern at least three animals. Significance levels for comparison between groups were determined with unpaired two-tailed Student's *t*-test were appropriate, using GraphPad Prism 6 (GraphPad Software) or Excel (Microsoft). Quantifications are plotted as mean±s.e.m. Unpaired Student's two-tailed *t*-tests were used, with significance levels *P<0.05, **P<0.01 and ***P<0.001. Use of other statistical tests is indicated in figure legends. Statistics from western blot analysis and *in vitro* studies are included in figure legends.

### Data availability

All relevant data are available from the authors.

## Additional information

**How to cite this article:** Jablonska, B. *et al*. Sirt1 regulates glial progenitor proliferation and regeneration in white matter after neonatal brain injury. *Nat. Commun.* **7,** 13866 doi: 10.1038/ncomms13866 (2016).

**Publisher's note:** Springer Nature remains neutral with regard to jurisdictional claims in published maps and institutional affiliations.

## Supplementary Material

Supplementary Information Supplementary Figures and Supplementary Tables.
