# Supplementary material for: Sirt1 regulates glial progenitor proliferation and regeneration in white matter after neonatal brain injury
Source: Nat Commun. 2016 Dec 19;7:13866. doi: 10.1038/ncomms13866 (PMC5187440; doi:10.1038/ncomms13866)
Supplement: Supplementary Information — Supplementary Figures and Supplementary Tables. [file ncomms13866-s1.pdf]

Supp. Figure 1 (Jablonska and Gallo)

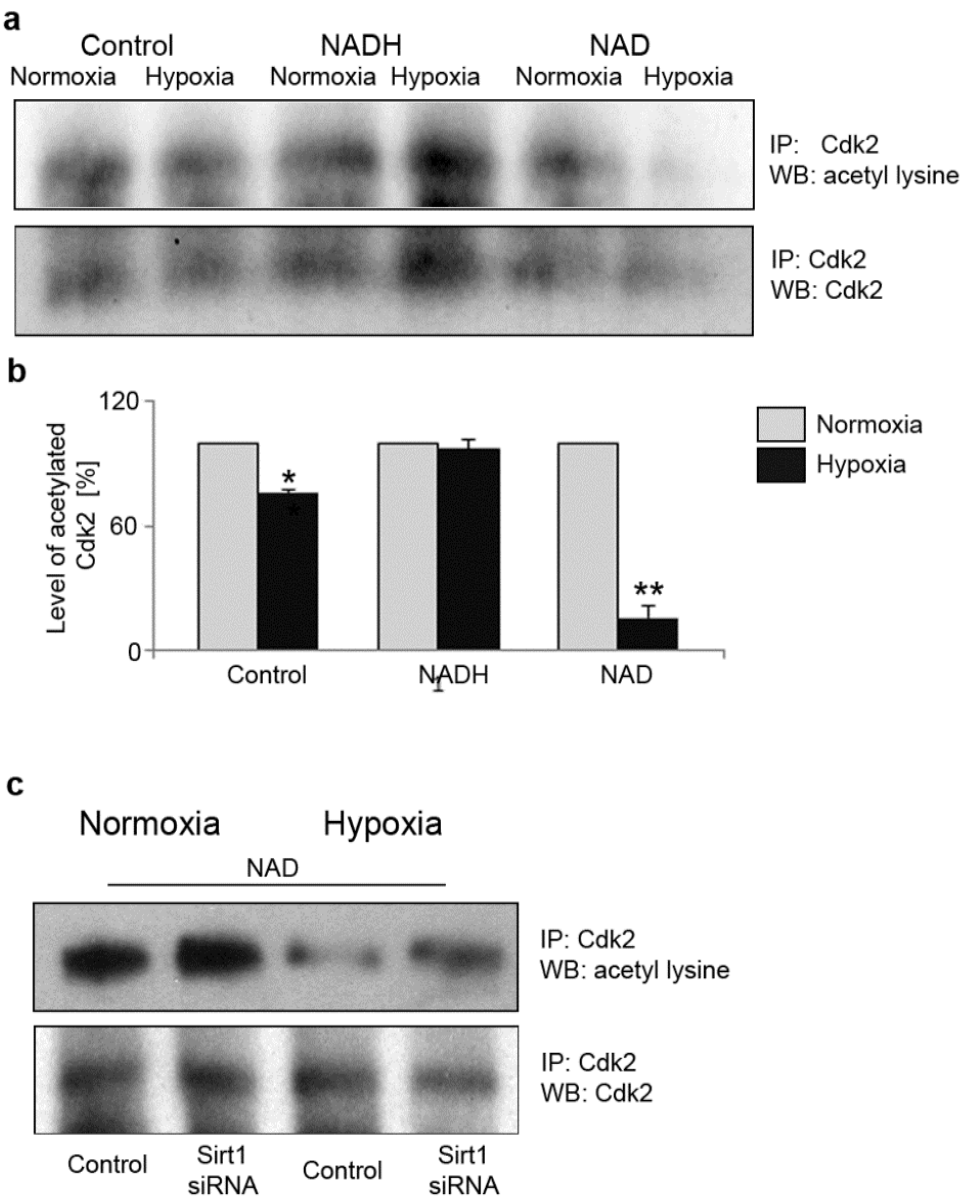

Supp. Figure 1. An increased level of NAD elevates Sirt1 activation in HX. Representative Western blots of immunoprecipitated Cdk2 from cultured white matter cells showing decreased acetylation with NAD specifically in HX (a). Graph represents levels of acetylated Cdk2 in NX and HX white matter lysates as a percentage of total Cdk2 levels. Elevating NAD levels causes significant reduction in Cdk2 acetylation after HX (b). (mean $\pm$ SEM, n=3 brains per condition). (c) Loss-of-function experiment on NX and HX cells exposed to NAD. IP-Western blot showed higher expression of acetylated-Cdk2 in NX and

Supp.Figure 2 (Jablonska and Gallo)

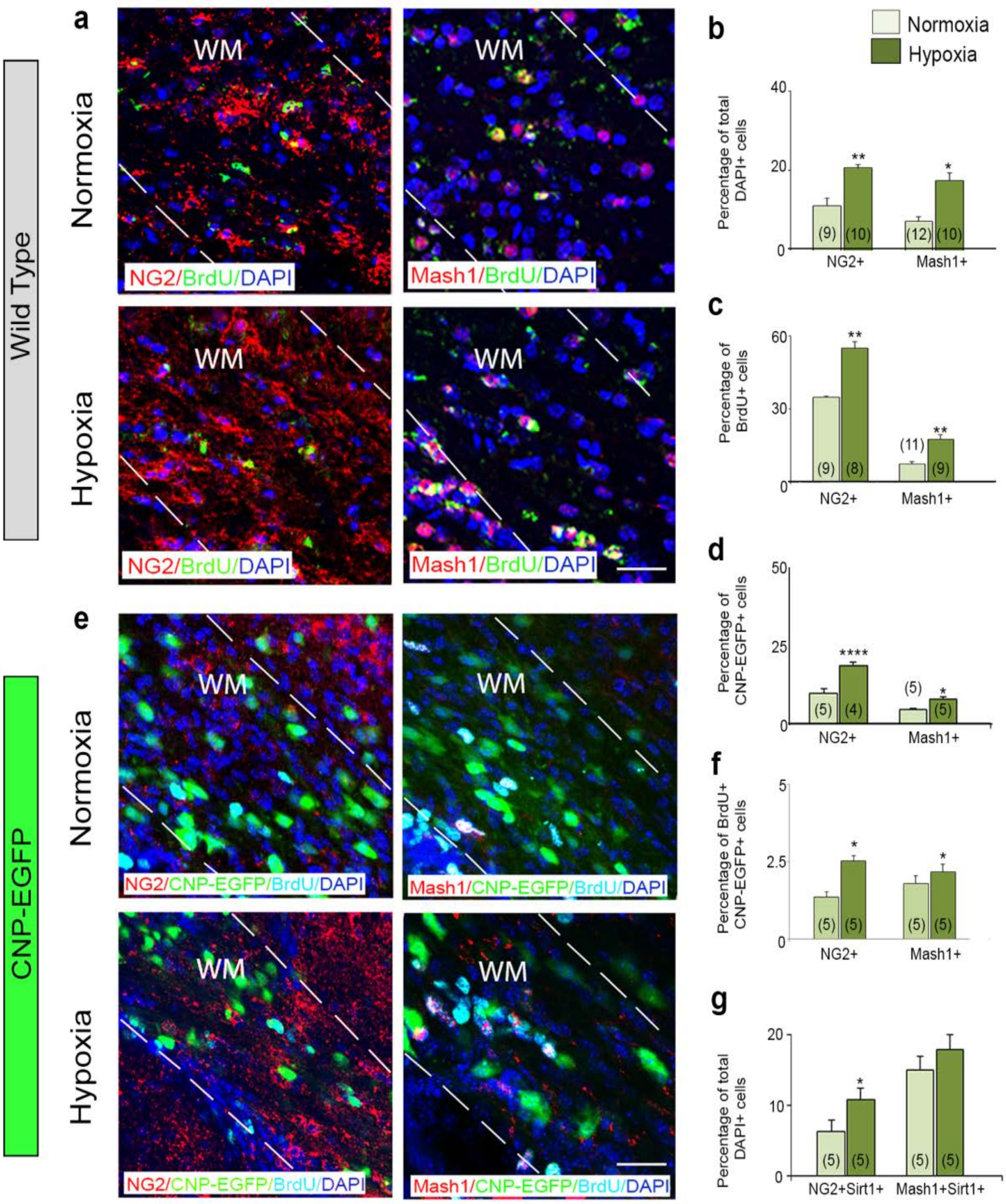

Supp. Figure 2. Neonatal HX enhances parenchymal OPC proliferation in the developing white matter. Confocal images of NX and HX white matter from WT (a) and CNP-EGFP mice (e) at P18. WM: white matter. Dotted lines delimit white matter. Scale bar = 100µm. Progenitor cells were stained with anti-NG2 or anti-Mash1 antibodies (b, d) and anti-Sirt1 antibody (g), or anti-BrdU antibodies (c, f). HX increases the percentage of $NG2^{+}$ and $Mash1^{+}$ progenitors, as well as their proliferation. HX also increases the percentages of $NG2^{+}Sirt1^{+}$ but not $Mash1^{+}Sirt1^{+}$ cells (g). Histograms show mean $\pm$ SEM. Number in parentheses within bar indicates number of samples, n=4 brains for each condition.

Supp. Figure 3 (Jablonska and Gallo)

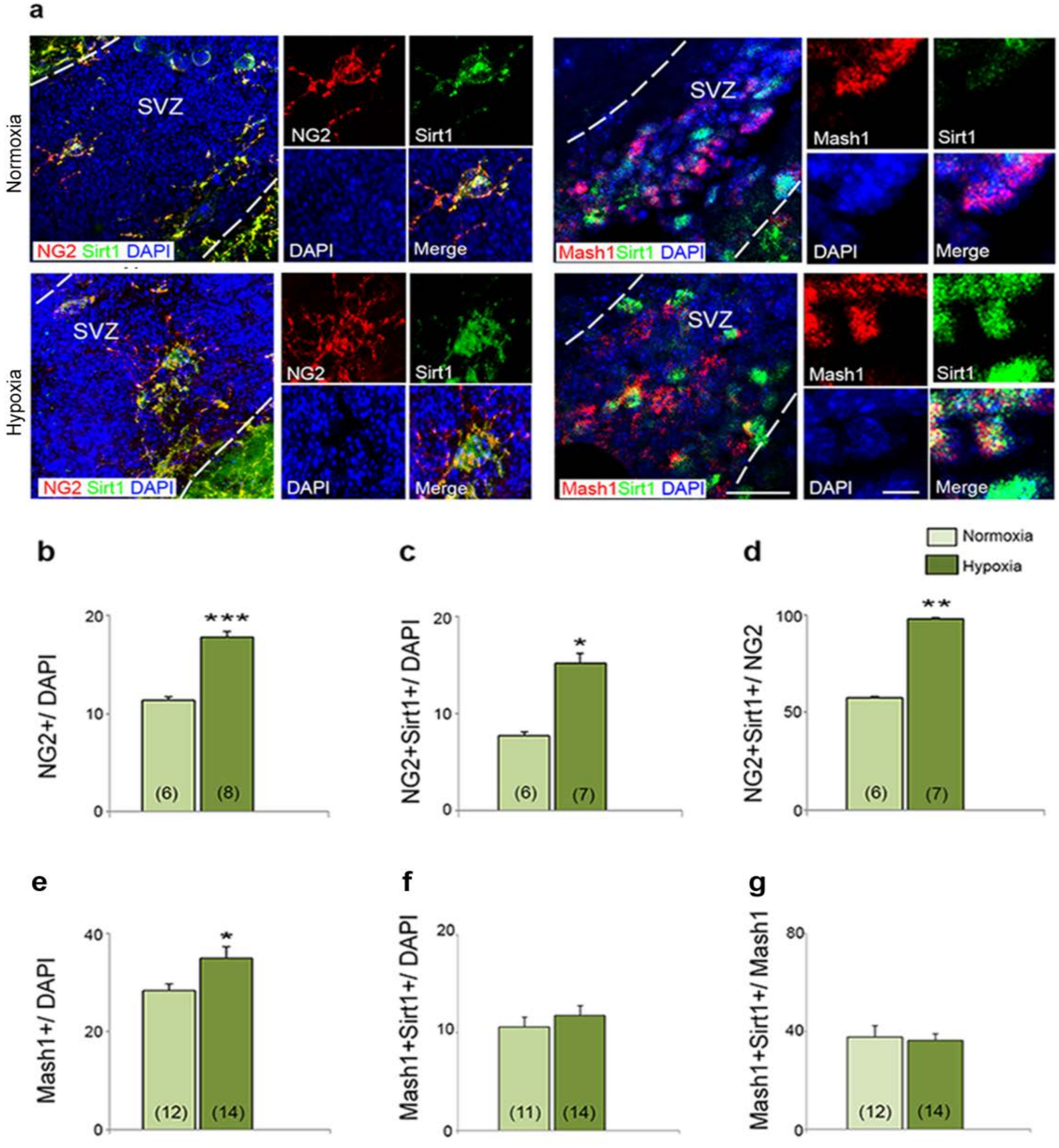

Supp. Figure 3. Neonatal HX increases NG2$^+$ cells in the SVZ. Confocal images of NG2$^+$ and Mash1$^+$ progenitor cells from NX and HX SVZ at P18 (a). SVZ: subventricular zone, dotted lines delimit SVZ. Scale bar= 100µm. Graphs present percentages of total NG2$^+$ (b) and Mash1$^+$ (e) cells, as well as NG2$^+$Sirt1$^+$ (c,d) and Mash1$^+$Sirt1$^+$ (f,g) cells. HX upregulates Sirt1 expression in SVZ NG2$^+$ progenitors, but not in SVZ Mash1$^+$ cells. Histograms show mean ± SEM. Number in parentheses within bar indicates number of samples, n=4 brains per condition for each antibody.

Supp. Figure 4 (Jablonska and Gallo)

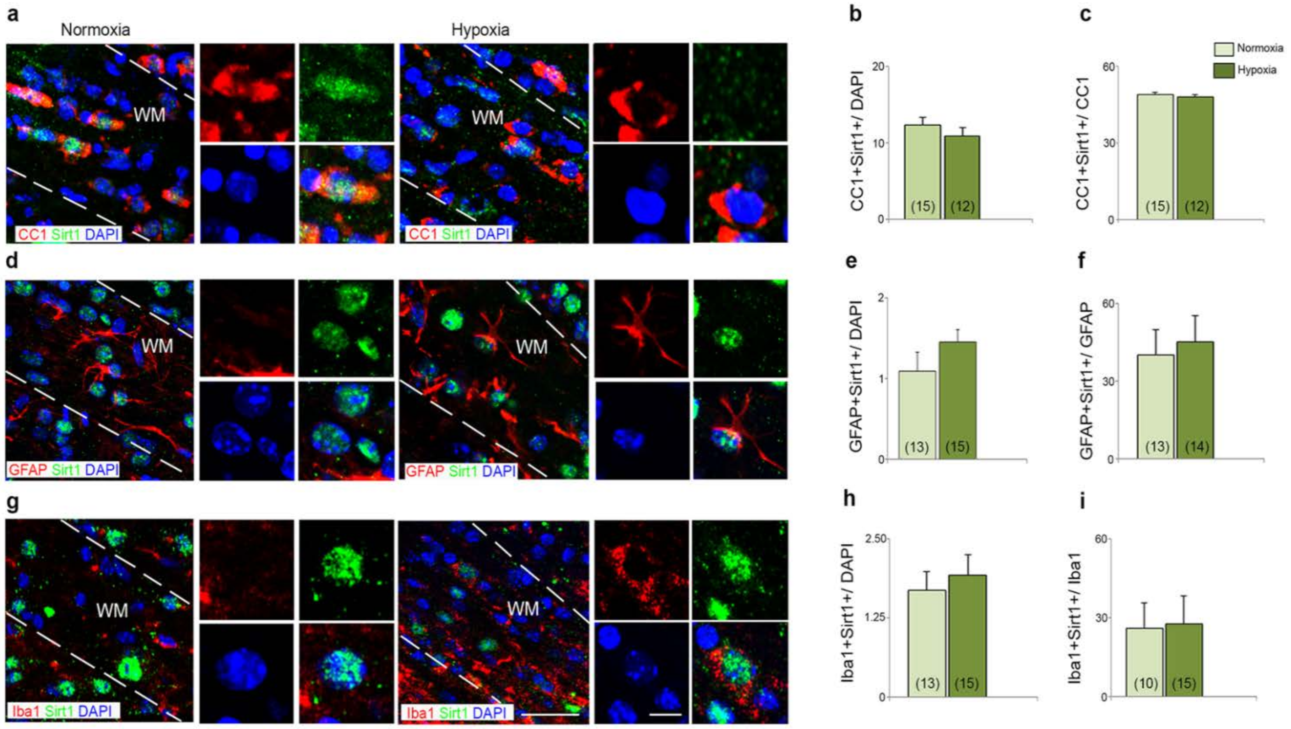

Supp. Figure 4. Sirt1 expression in various glia cell types in white matter. Confocal images from NX and HX white matter of WT mice at P18. WM: white matter. Dotted lines delineate white matter. Scale bar = 100µm. Cells were stained with anti-Sirt1 and co-labeled with anti-CC1 (a), anti-GFAP (d) and anti-Iba1 (g) antibodies. Inserts show an example of Sirt1 expression in a $CC1^+$, $GFAP^+$, and $Iba1^+$ at higher magnification. Graphs present the percentages of Sirt1 expressing mature OLs (b, c), astrocytes (e, f) and microglia (h, i). No changes in these cell types are observed after HX. Histograms show mean ± SEM. Number in parentheses within bar indicates number of samples, n=4 brains for each condition.

Supp. Figure 5 (Jablonska and Gallo)

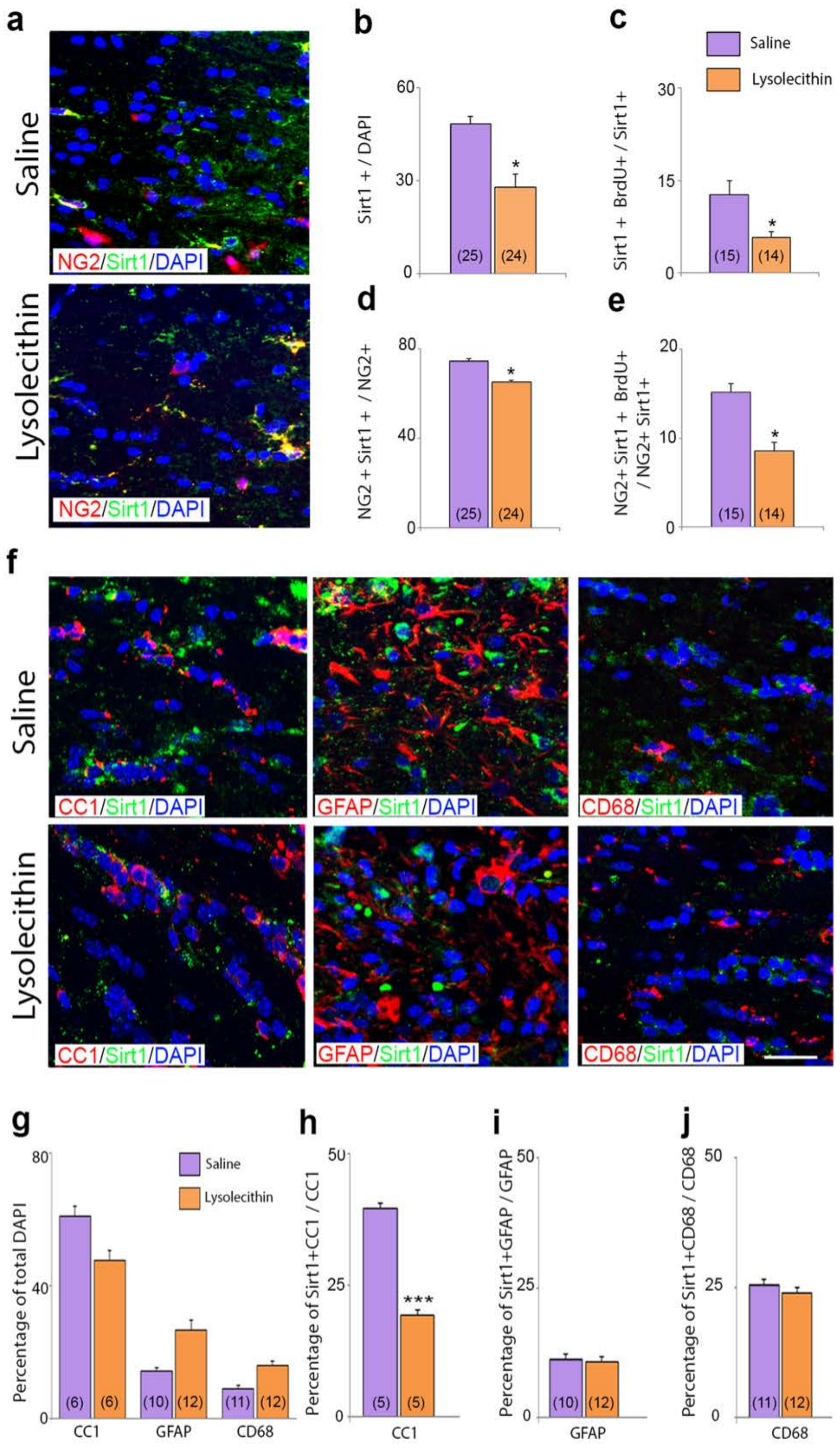

Supp. Figure 5. Demyelination exerts an opposite effect on Sirt1 expression in progenitor cells in white matter. Confocal images of Sirt1 distribution in control and demyelinated white matter at P60 (a, f). Scale bar = 100µm. Progenitor cells were labeled with anti-NG2 antibody, mature OLs with anti-CC1, astrocytes with anti-GFAP, and reactive microglia with anti-CD68 antibodies. Graphs present the percentage of Sirt1$^+$ cells (b), proliferating Sirt1$^+$ cells (c), NG2$^+$Sirt1$^+$ (d), and NG2$^+$Sirt1$^+$BrdU$^+$ (e) cells in white matter and , total percentage of glial cell types (g). The percentage of mature OLs expressing Sirt1 decreases (h), whereas the percentages of Sirt1$^+$GFAP$^+$ (i) and Sirt1$^+$CD68$^+$ (j) remain unchanged after HX. All histograms show mean $\pm$ SEM. Number in parentheses within bar indicates number of samples, n=5 brains per condition, and for each antibody.

Supp. Figure 6 (Jablonska and Gallo)

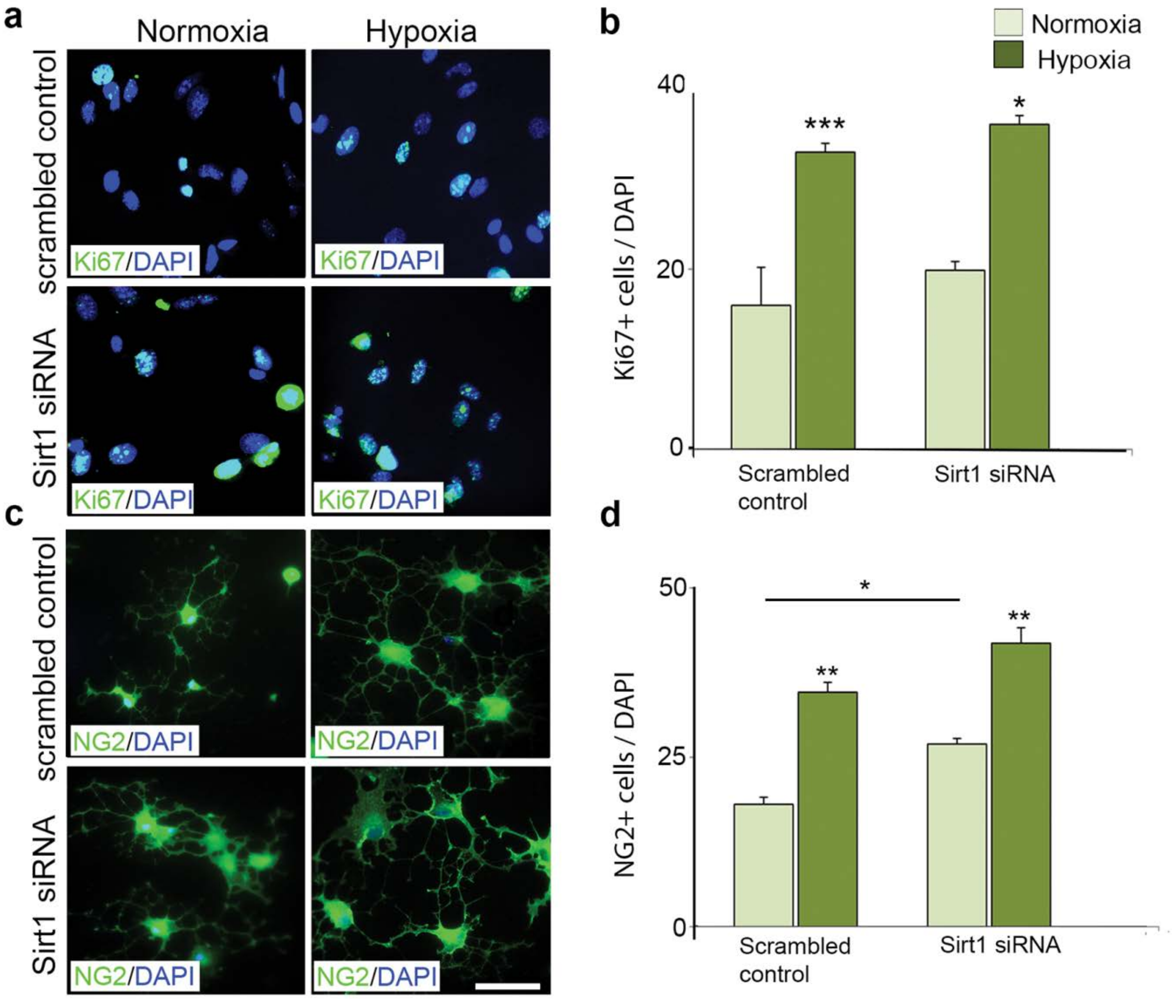

Supp. Figure 6. Inactivation of *Sirt1* in SVZ-derived OPCs does not alter the effects of neonatal HX on their proliferation. Cells isolated from NX and HX SVZ at P18 were transfected with Sirt1 siRNA and with scrambled control. OPC proliferation was assessed by double labeling with anti-Ki67 (a,b) and anti-NG2 (c,d). Scale bar = 100µm. Graphs represent the percentage of $Ki67^+$ (b) and $NG2^+$ (d) cells in NX and HX cultures treated with scrambled control and Sirt1 siRNA. In control and Sirt1 siRNA-transfected cultures, HX upregulates cell proliferation (b) and the percentage of $NG2^+$ progenitor cells (d). Five NX and 4 HX brains. Histograms show mean ± SEM of three cultures per condition.

Supp. Figure 7 (Jablonska and Gallo)

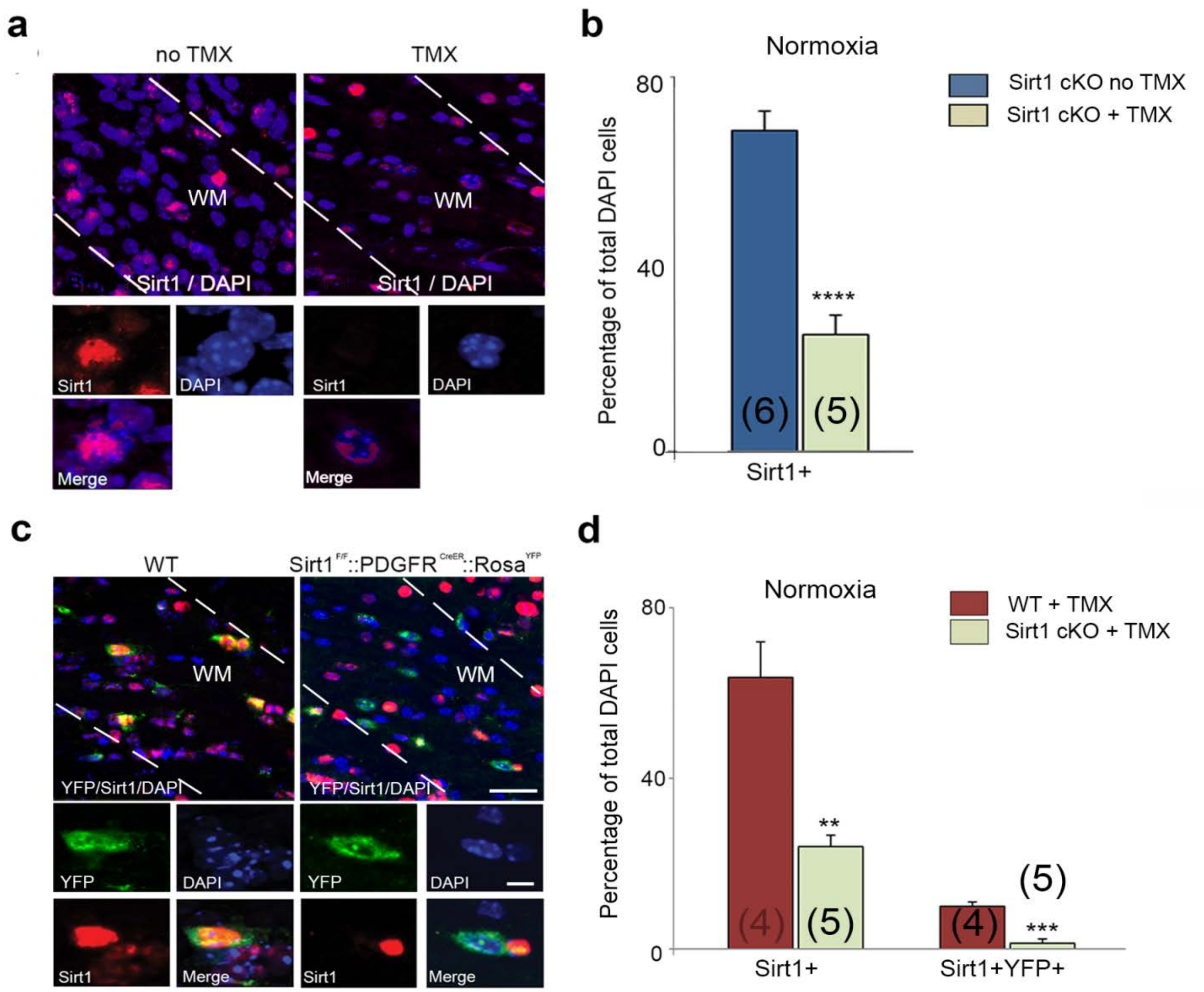

Supp. Figure 7. Characterization of cell proliferation in Sirt1$^{F/F}$;PDGFR^{CreER};Rosa^{YFP} mice under normal physiological conditions. Confocal images from white matter of NX and HX WT and Sirt1$^{F/F}$;PDGFR^{CreER};Rosa^{YFP} mice (a,c). Cells were stained with anti-Sirt1, anti-GFP (to detect YFP) antibodies and DAPI. Dotted lines delineate white matter. WM = white matter. Scale bar = 100µm. (b) Tamoxifen injection causes a reduction of total Sirt1^{+} cells in Sirt1$^{F/F}$;PDGFR^{CreER};Rosa^{YFP} mice as compared to the same genotype without TMX (b), as well as to their WT littermates (d). The percentage of Sirt1^{+}YFP^{+} cells is reduced in white matter of Sirt1$^{F/F}$;PDGFR^{CreER};Rosa^{YFP} mice (d). Number in parentheses within bar indicates number of samples. Histograms show mean $\pm$ SEM, n=3 brains per condition, for each antibody.

Supp. Figure 8 (Jablonska and Gallo)

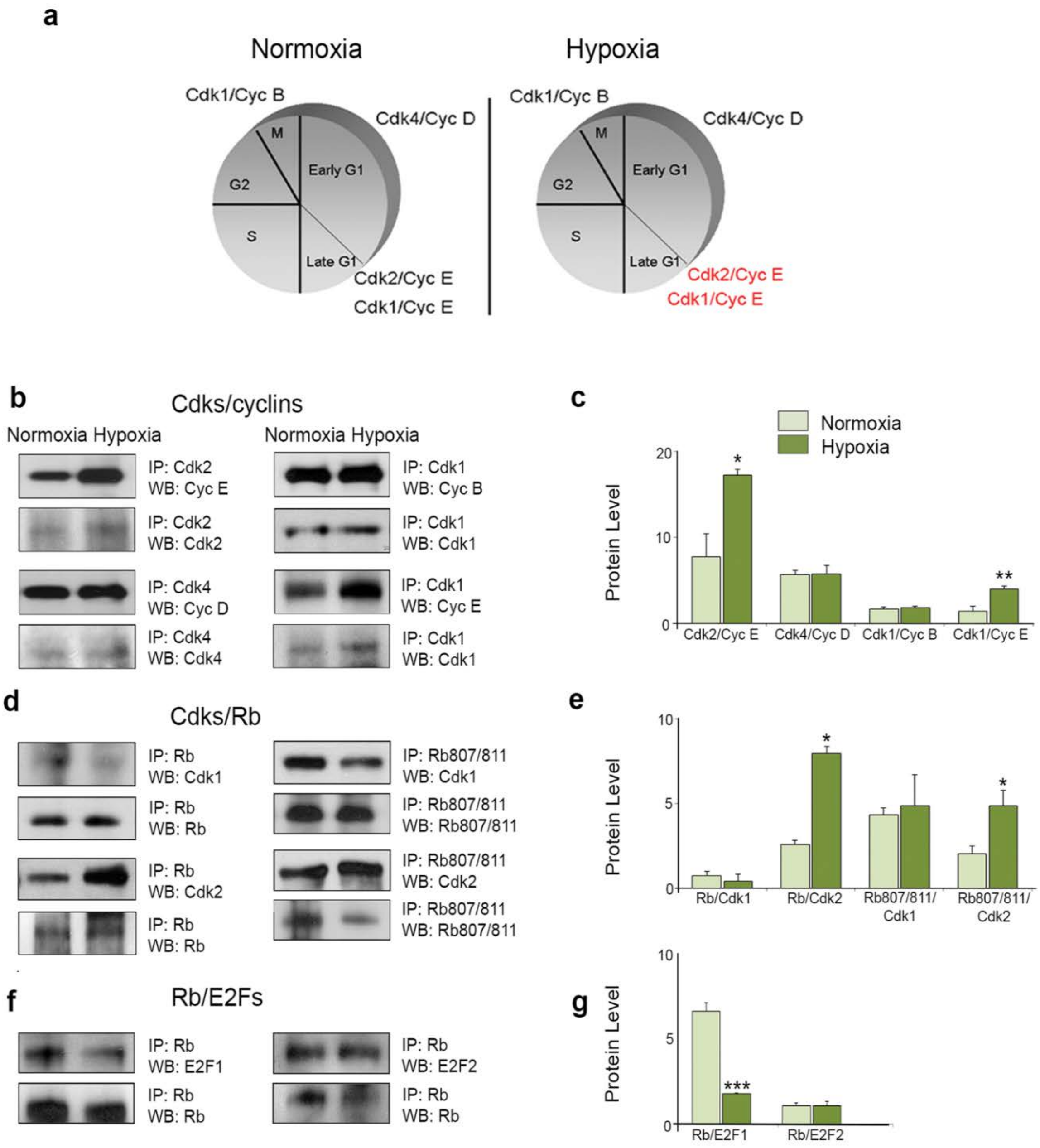

Supp. Figure 8. Neonatal HX induces Cdk2 activation in white matter. (a) Schematic drawing of the main Cdks/Cyclins complexes involved in cell cycle progression showing in red complexes whose expression is enhanced by HX. (b) Representative images showing biochemical analysis of Cdks/Cyclins (b), Cdks/Rb (d) and Rb/E2Fs (f) complexes in NX and HX white matter (P18) analyzed by IP and Western blot. (c,e,g) Graphs represent relative protein levels of these complexes in NX and HX white matter. HX increases Cdk2/CycE, Cdk1/CycE, Rb/Cdk2, and pRb(807/811)/Cdk2 complex formation, whereas expression of the Rb/E2F1 complex is reduced. (mean + SEM, n=3 brains for each group, actin serves as control)

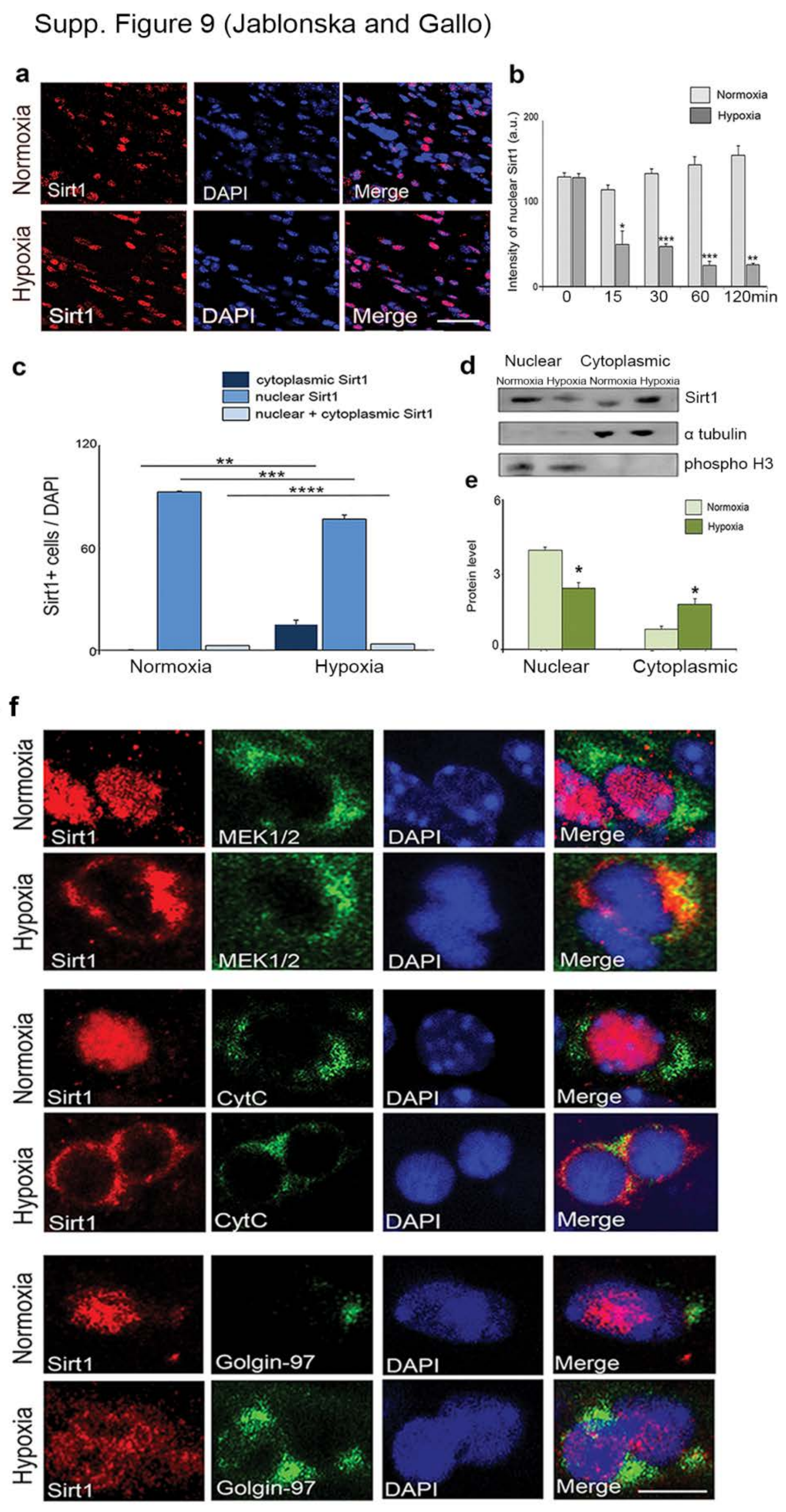

Supp. Figure 9. Neonatal HX induces Sirt1 translocation to cytoplasm. (a) Confocal images of white matter demonstrate predominantly nuclear intensity of Sirt1 staining in NX, which decreases after HX. (b) Intensity of Sirt1 staining is expressed in arbitrary units (a.u.) immediately (0 min), or 15, 30, 60, and 120 min after HX (Mean + SEM, n=4 brains for each condition, for each time point). (c) Graph represents percentage of Sirt1^{+} cells displaying cytoplasmic, nuclear, or nucleo-cytoplasmic Sirt1 in NX and HX (Mean + SEM, 2 NX, 6 HX brains). (d) Representative Western blot of subcellular fractions from white matter. Sirt1 is predominantly found in the nuclear fraction in NX, and in cytoplasm after HX. Alpha-tubulin and phospho-histone3 serves as controls for each fraction (n=3 brains for each condition). (e) Graph represents lower Sirt1 expression in nucleus and higher Sirt1 level in cytoplasm after HX. Mean + SEM (2 NX and 6 HX brains). (f) Localization of Sirt1 in cellular compartments co-stained with anti-MEK 1/2, -CytC, and -Glogin-97 antibodies demonstrates that HX increases Sirt1 expression in cytoplasm and mitochondria.

Supp. Figure 10 (Jablonska and Gallo)

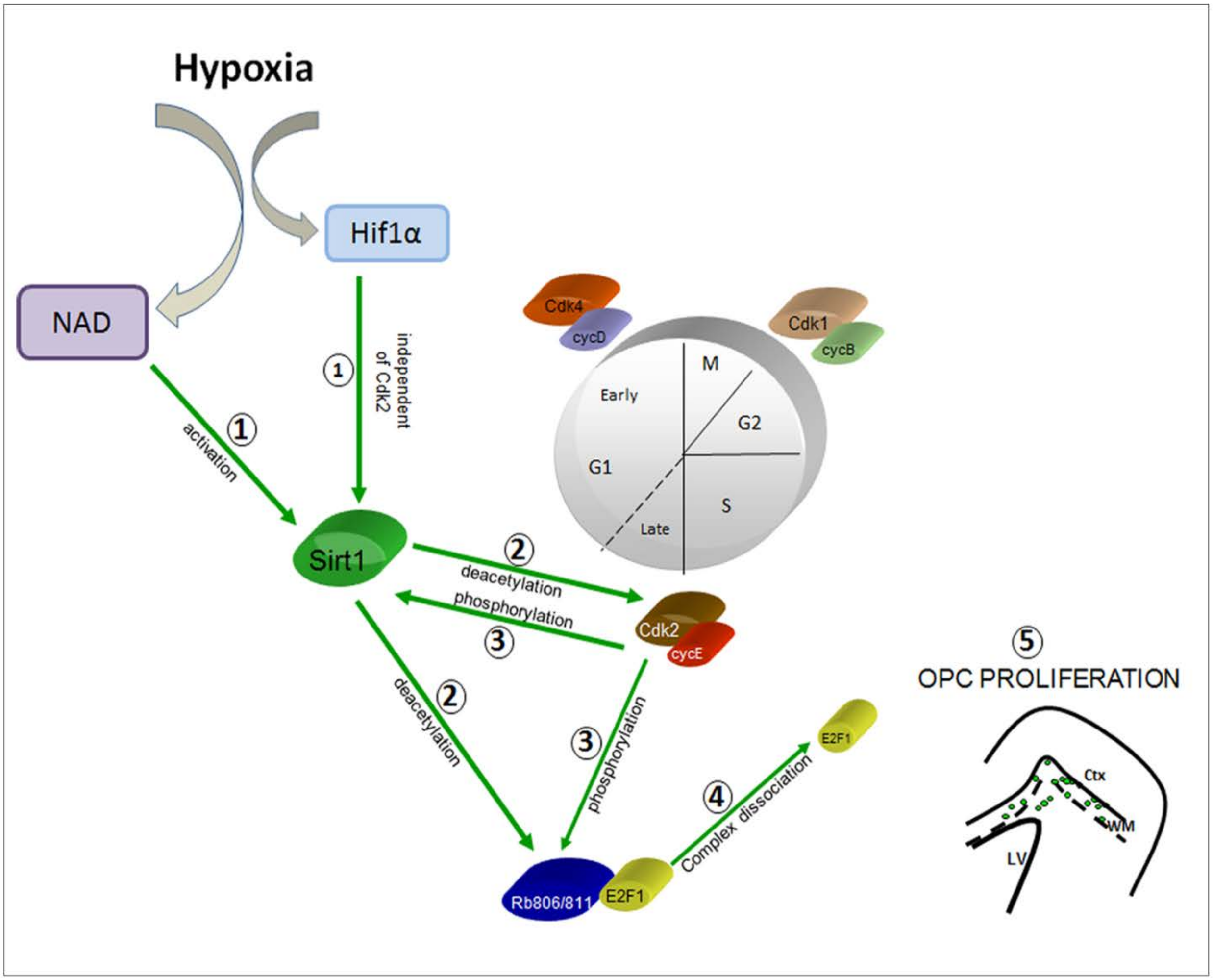

Supp. Figure 10. Mechanism of Sirt1-induced OPC proliferation after neonatal HX. Illustration of the molecular events involved in Sirt1-mediated OPC proliferation induced by HX. HX increases levels of Hif1α and NAD, which respectively enhance Sirt1 expression and activity. Activated Sirt1 deacetylates Cdk2 and Rb. The deacetylation of Cdk2 leads to Sirt1 phosphorylation at Ser47. Rb deacetylation by Sirt1 and its phosphorylation by Cdk2 causes dissociation of the Rb/E2F1 complex and release of the E2F1 transcription factor. Higher levels of unbound E2F1 promote OPC cell cycle entry and maintain their proliferative state.

Supp. Figure 11 (Jablonska and Gallo)

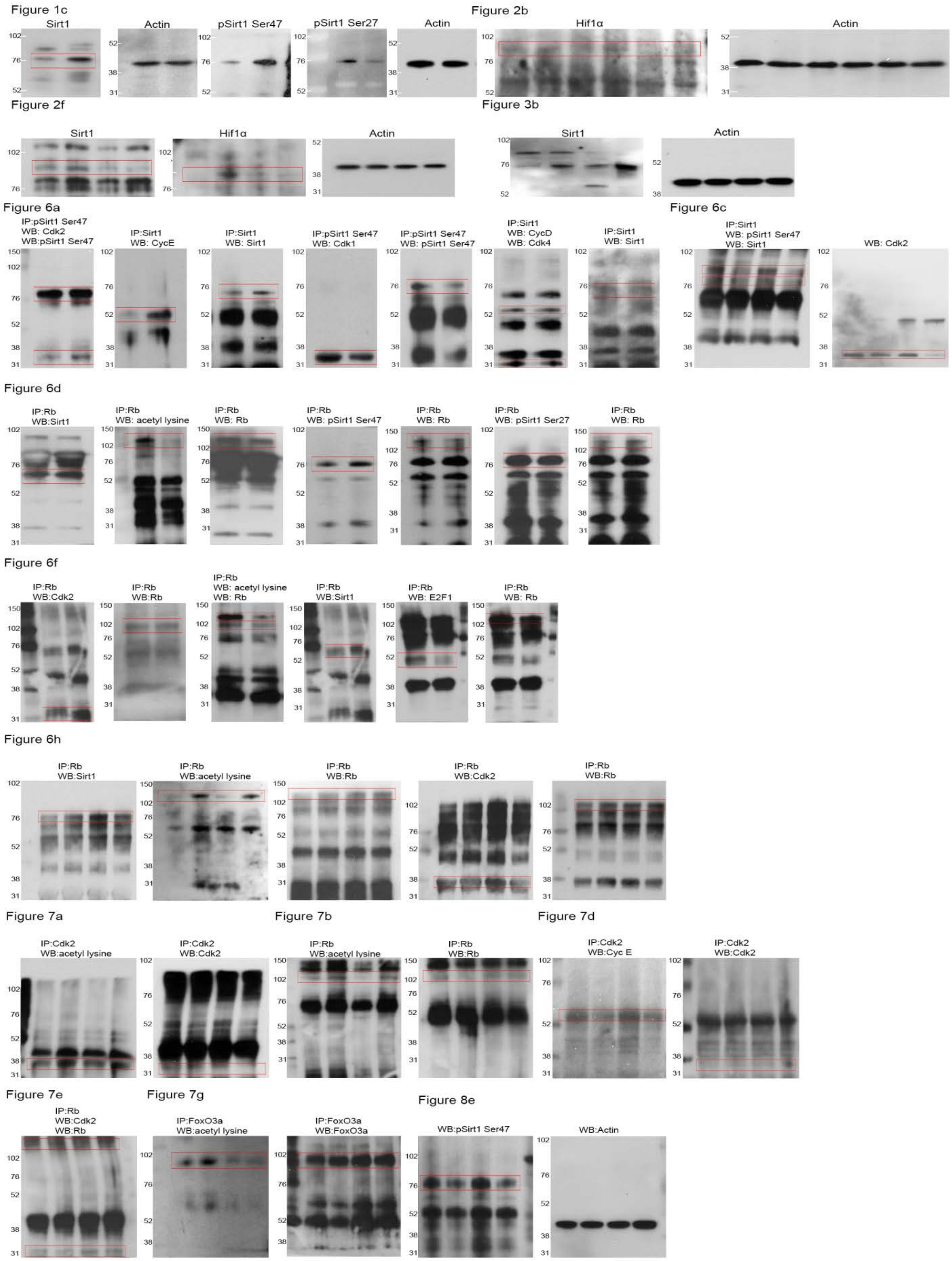

Supp. Figure 11. Full Western blots for all representative images shown in Main Figures. Red boxes indicate bands shown in Main Figures. Molecular weight marker is shown to the left of all images.

Supp. Figure 12 (Jablonska and Gallo)

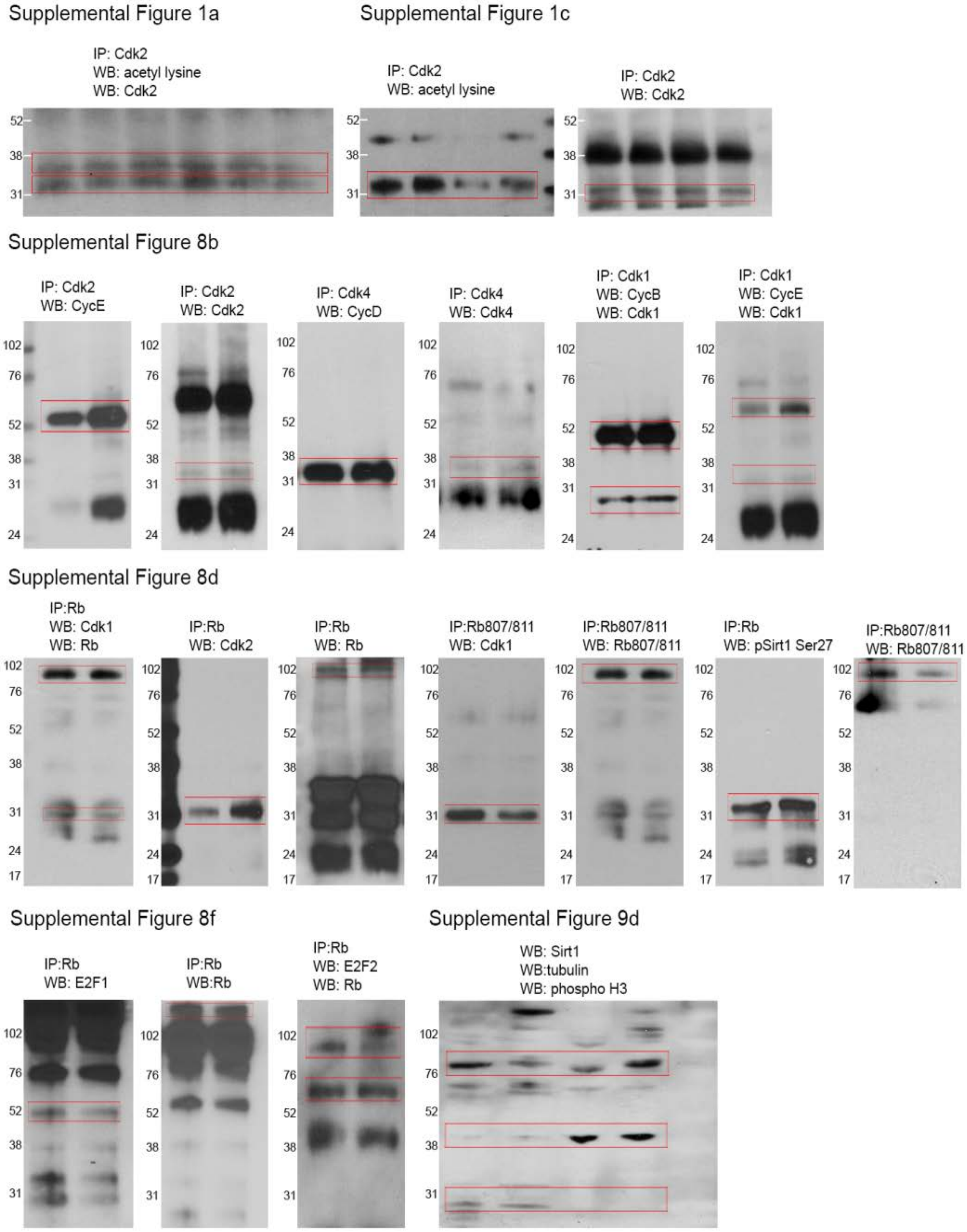

Supp. Figure 12. Full Western blots for all representative images shown in Supplemental Figures. Red boxes indicate bands shown in Main Figures. Molecular weight marker is shown to the left of all images.

Supplementary Information

Supplementary Table 1. RT-PCR Primers

Gene	Forward	Reverse
NG2	CGT GATG GTG TCT TTC GAT G	GAG TAC ATC ATG CCG ACT GC
Sirt1	GGT TGA CTT AGG TCT TGT CTG	CGT CCC TTG TAA TGT TTC CC
Olig2	CAA GTC ATC TTC CTC CAG CAC	GTA GAT CTC CTC GCT CAC CAG TCG
CNP	CCG GAG ACA TAG TGC CCG CA	AAA GCT GGT CCA GCC GTT CC
MBP	CCC TCT CTC CTG TTT CAG TG	CTA CCC ACT GTC GAT GAC TTA TTG ATT AGA G
GFAP	ACTTAACAAATCCCTTCCTTCATCC	CCC TCT CTC CTG TTT CAG TG
Actin	CGT GGG CCG CCC TAG GCA CCA	TTG GCC TTA GGG TTC AGG GGG
